# *Phytophthora* Have Distinct Endogenous Small RNA Populations That Include Short Interfering and microRNAs

**DOI:** 10.1371/journal.pone.0077181

**Published:** 2013-10-21

**Authors:** Noah Fahlgren, Stephanie R. Bollmann, Kristin D. Kasschau, Josh T. Cuperus, Caroline M. Press, Christopher M. Sullivan, Elisabeth J. Chapman, J. Steen Hoyer, Kerrigan B. Gilbert, Niklaus J. Grünwald, James C. Carrington

**Affiliations:** 1 Donald Danforth Plant Science Center, St. Louis, Missouri, United States of America; 2 Center for Genome Research and Biocomputing, Department of Botany and Plant Pathology, Oregon State University, Corvallis, Oregon, United States of America; 3 Horticultural Crops Research Laboratory, USDA Agricultural Research Service, Corvallis, Oregon, United States of America; Universidade Federal do Rio Grande do Sul, Brazil

## Abstract

In eukaryotes, RNA silencing pathways utilize 20-30-nucleotide small RNAs to regulate gene expression, specify and maintain chromatin structure, and repress viruses and mobile genetic elements. RNA silencing was likely present in the common ancestor of modern eukaryotes, but most research has focused on plant and animal RNA silencing systems. *Phytophthora* species belong to a phylogenetically distinct group of economically important plant pathogens that cause billions of dollars in yield losses annually as well as ecologically devastating outbreaks. We analyzed the small RNA-generating components of the genomes of *P. infestans, P. sojae* and *P. ramorum* using bioinformatics, genetic, phylogenetic and high-throughput sequencing-based methods. Each species produces two distinct populations of small RNAs that are predominantly 21- or 25-nucleotides long. The 25-nucleotide small RNAs were primarily derived from loci encoding transposable elements and we propose that these small RNAs define a pathway of short-interfering RNAs that silence repetitive genetic elements. The 21-nucleotide small RNAs were primarily derived from inverted repeats, including a novel microRNA family that is conserved among the three species, and several gene families, including Crinkler effectors and type III fibronectins. The *Phytophthora* microRNA is predicted to target a family of amino acid/auxin permeases, and we propose that 21-nucleotide small RNAs function at the post-transcriptional level. The functional significance of microRNA-guided regulation of amino acid/auxin permeases and the association of 21-nucleotide small RNAs with Crinkler effectors remains unclear, but this work provides a framework for testing the role of small RNAs in *Phytophthora* biology and pathogenesis in future work.

## Introduction

*Phytophthora* species are a diverse group of filamentous, eukaryotic plant pathogens that are related to other stramenopile (heterokont) eukaryotes within the chromalveolate super-group [1], [2]. The stramenopile group includes golden-brown algae, diatoms, brown algae, and oomycetes. These diverse organisms, ranging from autotrophic algae to pathogenic, fungus-like oomycetes, were only recently grouped together based on phylogenetic analysis [1]. The oomycete lineage (non-photosynthetic stramenopiles, including the genus *Phytophthora*) was shown to be evolutionarily ancient. The oomycota lineage is estimated to have split from the photosynthetic stramenopiles in the ochrophyta lineage near the Proterozoic-Phanerozoic boundary ca. 570 million years ago [2]. The genus *Phytophthora* contains some of the most devastating plant pathogens and contains upwards of 100 formally described species [3]. *Phytophthora* species are capable of infecting nearly all eudicot plants and several monocot species, causing multibillion-dollar damage to crops, ornamental plants, and natural environments. *P. infestans* infection of potatoes and tomatoes causes late blight disease and is best known as the cause of the Irish potato famine [4], [5]. *P. sojae* is an economically important pathogen of soybeans resulting in estimated losses of $1–2 billion annually worldwide [6]. *P. ramorum* causes sudden oak death and infects a large variety of ornamental nursery crops [7]. *P. ramorum* infections have afflicted trees in the western United States since the mid-1990s and more recently gained notoriety for causing massive landscape-scale dieback in plantations of Japanese larch in the United Kingdom [8]. The genomes of these three *Phytophthora* species have been sequenced [9], [10], thus enabling discovery of the silencing machinery employed and the resulting small RNA expression patterns observed in this group. The fact that the oomycete lineage is ancient and basal to the stramenopile group provides an opportunity to elucidate the evolutionary history of the silencing machinery in the stramenopiles.

Eukaryotes use RNA-based pathways to regulate gene expression at the transcriptional and post-transcriptional levels. RNA silencing pathways utilize small RNAs (∼20–30 nucleotides) to program effector protein complexes to recognize specific target nucleic acids in a sequence-dependent manner [11]. Target recognition by small RNA-effector complexes generally results in the suppression of activity of the target, either through degradation or recruitment of additional silencing factors. Generally, small RNAs are generated from double-stranded RNA through the activity of one or more Ribonuclease III (RNaseIII)-like enzymes [12]. Therefore, the conversion of single-stranded RNA into double-stranded RNA is a key step in RNA silencing pathways and occurs by intramolecular hybridization of self-complementary RNA to form a stem-loop (or hairpin) RNA, or by conversion of RNA into double-stranded RNA through the action of an RNA-dependent RNA polymerase (RDR). Several classes of small RNA are distinguished and include most notably the short-interfering RNAs (siRNA) and microRNAs (miRNA).

In plants and animals, and several other eukaryotic lineages, microRNAs are ∼20-24-nucleotide RNAs that mediate silencing of target transcripts post-transcriptionally. Primary miRNA transcripts (pri-miRNA) are generally transcribed by RNA polymerase II and contain self-complementary regions that fold to form imperfect double-stranded stem-loop structures. In animals, pri-miRNAs are initially processed by the RNaseIII protein DROSHA, or by the spliceosome in the case of mirtrons, to form precursor-miRNA (pre-miRNA). The miRNA and duplex strand (miRNA*) are released from the stem-loop RNA by the RNaseIII protein DICER (DCR) [13]. Plants lack a DROSHA homolog and instead DICER-LIKE1 (DCL1), a homolog of DCR, catalyzes both miRNA-processing steps [14]. The resulting miRNA/miRNA* duplex has 5′ monophosphates and 2-nucleotide, 3′ overhangs [15]. The final maturation step requires proper selection of the miRNA (guide strand) from the miRNA/miRNA* duplex by a member of the ARGONAUTE (AGO) protein family. AGOs select guide strands by one or more criteria including the miRNA 5′ end thermodynamic stability, 5′ nucleotide identity, miRNA/miRNA* duplex structure and in some cases other sequence-specific interactions [16]. AGO proteins loaded with miRNA are programmed to recognize and repress specific cellular target RNAs through AGO-mediated cleavage or translational repression [11]. MiRNA-guided target repression is an important regulator of developmental timing and patterning and response to biotic and abiotic signals [15], [17].

Another diverse class of small RNAs, referred to broadly as siRNA, are common to animals, plants, and fungi [18], [19]. Unlike miRNAs, siRNAs are generally produced from fully complementary RNA, generated through the activity of an RDR, or in some cases long, hairpin RNAs with nearly full complementarity. Biogenesis and function of siRNAs are diverse and include adaptive defense against viruses, maintenance of genome stability and chromatin structure, identification and suppression or elimination of transposable elements and duplicated DNA, and transcriptional and post-transcriptional control of gene expression [20], [21], [22], [23]. In many lineages, the diversification of small RNA pathways has been accompanied by duplication and diversification of small RNA biogenesis factors (DCR/DCL and RDR) and effectors (AGO) [24].

Small RNAs have been studied in several pathosystems [25], [26], [27], [28], [29], but their role in regulating pathogen biology and host-pathogen interactions is poorly understood. Endogenous silencing pathways and differential expression of different classes of small RNAs during host-pathogen interaction remain to be characterized for most pathogens, including *Phytophthora* species. *Dcl*, *Rdr* and *Ago* genes were described in *P. infestans* [30], and several small RNA size classes between 21- and 32-nucleotides in length were described by deep sequencing in *P. infestans* [28], but further work is needed to characterize and assess the conservation of RNA silencing pathways in the *Phytophthora* genus. We used deep sequencing and comparative genomic approaches to analyze the endogenous small RNA populations of *P. infestans, P. ramorum* and *P. sojae.* A bimodal distribution of small RNA sizes was observed across all three species with peaks centered on 21- and 25-nucleotide long small RNAs. A bimodal distribution is suggestive of separate processing by the two DCL homologs in each species. The larger small RNA size class is predominantly associated with loci encoding transposable elements or other repetitive DNA while the shorter class was associated with several gene families, including Crinkler effectors and type III fibronectins, inverted repeats and a novel, conserved miRNA family. This work is a crucial step in elucidating the endogenous RNA silencing pathways in these important plant pathogens and provides novel insights into the silencing machinery in the stramenopiles.

## Results/Discussion

### RNA Silencing Machinery in Phytophthora Species

**Figure 1:**
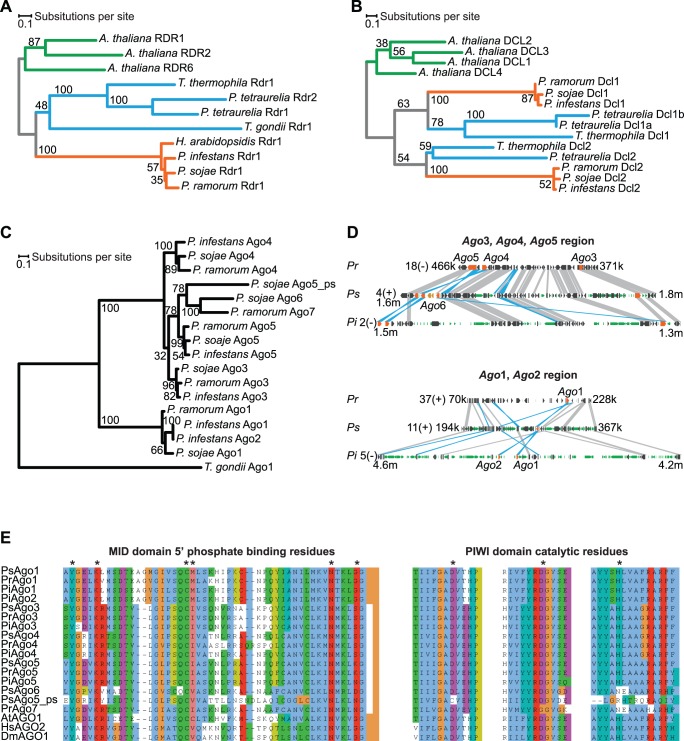
RNA silencing machinery in *Phytophthora*. (A–C) Phylogenetic analysis of Rdr, Dcl and Ago proteins, respectively. Protein domains used for analysis were the Rdr RdRP domain, the Dcl RNaseIII A and B domains and the Ago DUF1785-PAZ-PIWI region. Maximum Likelihood bootstrap support values are shown. In (A) and (B), taxonomic groups are color-coded: viridiplantae (green), alveolates (blue) and oomycetes (orange). (D) Synteny maps of regions containing Ago3–5 (top) and Ago1/2 (bottom) in *P. ramorum* (*Pr*), *P. sojae* (*Ps*), and *P. infestans* (*Pi*). Regions are labeled with scaffold numbers, alignment orientation (+/−) and coordinate ranges. Genes (dark arrows), Ago genes (orange arrows), and transposable elements (green arrows) are depicted. Conserved intervals are connected by gray (direct) or blue (inverse) boxes (E) MAFFT alignment with ClustalX coloring of amino acid residues from the Ago MID (5′ phosphate binding) and PIWI (catalytic) domains. Positions shown to interact with the guide-strand 5′ monophosphate (MID), and the Mg^2+^-coordinating catalytic residues (PIWI) are marked with an asterisk [40], [41], [42], [43], [44], [45], [46].

RNA interference (RNAi) is an established tool for studying gene function in *Phytophthora* species [31], [32], [33], [34], [35], [36], [37], [38], [39], which indicates that small RNA-based pathways exist in *Phytophthora*. In agreement with the observed activity of RNAi, homologs of the core eukaryotic RNA silencing machinery were identified in *P. infestans*, including one *Dcl*, one *Rdr*, and five *Ago* genes [30]. To determine if this complement of core silencing machinery is common among *Phytophthora* species, we used the genomic resources available for *P. infestans*, *P. sojae*, and *P. ramorum* to identify homologs in the three phylogenetically diverse species (see methods) [3], [9], [10]. Each *Phytophthora* genome encodes a single Rdr protein (Table 1). Phylogenetic analysis of the signature RNA-dependent RNA Polymerase (RdRP) domain suggests that the common ancestor of oomycetes (and possibly chromalveolates) had a single *Rdr* gene (Figure 1A). Two *Dcl* genes were found in *P. sojae* and *P. ramorum*, but *P. infestans* was previously reported to have a single *Dcl* gene [30], *PiDcl*1, that is orthologous to *PsDcl*1 and *PrDcl*1 (Table 1). We hypothesized that *PiDcl*2 was either lost or was mistakenly excluded from the *P. infestans* genome assembly. To address the latter possibility, we searched for evidence of *PiDcl*2 in the whole-genome shotgun reads from *P. infestans* at the National Center for Biotechnology Information (NCBI) Trace Archive. We were able to assemble a 3962-nucleotide contig that contained a *Dcl* gene that was distinct from *PiDcl*1 and shared significant amino acid similarity to PsDcl2 and PrDcl2, confirming that *P. infestans* has two *Dcl* genes (Figure 1B, Table 1). Dcl1 and Dcl2 from *Phytophthora* species and the alveolates *Tetrahymena thermophila* and *Paramecium tetraurelia* are phylogenetically distinct, suggesting an ancient divergence (Figure 1B).

**Table 1:** *Dcl*, *Rdr*, *Ago* and *MIRNA* genes in three *Phytophthora* species.

Group	Locus	Locus accession	Position
*Rdr*1	*PiRdr*1	PITG_10457	Supercontig 18∶1359241–1367325+
*Rdr*1	*PsRdr*1	estExt_Genewise1Plus.C_3_t40457	Scaffold 3∶2028009–2036275+
*Rdr*1	*PrRdr*1	N/A^1^	Scaffold 67∶79140–87321−
*Dcl*1	*PiDcl*1	PITG_09292	Supercontig 14∶1931317–1936311+
*Dcl*1	*PsDcl*1	gm1.19145_g	Scaffold 10∶1947580–1952607−
*Dcl*1	*PrDcl*1	C_scaffold_46000013	Scaffold 46∶152941–157809−
*Dcl*2	*PiDcl*2	N/A^1^	N/A^1^
*Dcl*2	*PsDcl*2	fgenesh1_pg.4_#_534	Scaffold 4∶2583558–2586463−
*Dcl*2	*PrDcl*2	fgenesh1_pg.C_scaffold_43000060	Scaffold 43∶164754–168440−
*Ago*1	*PiAgo*1	PITG_04470	Supercontig 5∶4373459–4376242−
*Ago*1	*PiAgo*2^2^	PITG_04471	Supercontig 5∶4396571–4399354−
*Ago*1	*PsAgo*1	Physo2.s_453613	Scaffold 11∶289545–292340−
*Ago*1	*PrAgo*1	C_scaffold_37000014	Scaffold 37∶196057–199708+
*Ago*3	*PiAgo*3	PITG_01400	Supercontig 2∶1325116–1328925−
*Ago*3	*PsAgo*3	fgenesh1_pg.4_#_308	Scaffold 4∶1735957–1739823+
*Ago*3	*PrAgo*3	N/A^1^	Scaffold 18∶380881–384807−
*Ago*4	*PiAgo*4	PITG_01443	Supercontig 2∶1512873–1515521−
*Ago*4	*PsAgo*4	e_gw1.4.3046.1	Scaffold 4∶1590683–1593337−
*Ago*4	*PrAgo*4	fgenesh1_pg.C_scaffold_18000159	Scaffold 18∶445984–449397−
*Ago*5	*PiAgo*5	PITG_01444	Supercontig 2∶1518134–1520629−
*Ago*5	*PsAgo*5	fgenesh1_pm.4_#_266	Scaffold 4∶1586191–1588686−
*Ago*5	*PrAgo*5	C_scaffold_18000063	Scaffold 18∶453298–459311+
*Ago*6	*PsAgo*6	fgenesh1_pg.4_#_282	Scaffold 4∶1601071–1603176−
*Ago*7	*PrAgo*7	N/A^1^	Scaffold 104∶81401–85778+
*MIR*8788	*pin-MIR*8788	N/A^1^	Supercontig 18∶1064102–1064237−
*MIR*8788	*psj-MIR*8788a	N/A^1^	Scaffold 3∶1772538–1772675−
*MIR*8788	*psj-MIR*8788b	N/A^1^	Scaffold 3∶1777195–1777334+
*MIR*8788	*pra-MIR*8788a	N/A^1^	Scaffold 67∶213512–213649−
*MIR*8788	*pra-MIR*8788b	N/A^1^	Scaffold 1330∶2306–2443+

1 See File S2 for sequences.

2 *PiAgo*1 and *PiAgo*2 are co-orthologs with *PsAgo*1 and *PrAgo*1, therefore, the name *Ago*2 was not assigned to any gene in *P. sojae* or *P. ramorum*.

Five *Ago* homologs and six probable *Ago* pseudogenes were identified from all three *Phytophthora* species (Table 1, Table 2). In a phylogenetic analysis of the DUF1785, PAZ and RNaseH-like PIWI domain, *Phytophthora* Ago proteins formed two deeply branching clades (Figure 1C). One clade contained proteins encoded by the orthologous *Ago*1 genes and *PiAgo*2, an *Ago*1 paralog (Figure 1C and D). The second clade contained four closely related groups of Ago proteins (Figure 1C). The Ago3, Ago4 and Ago5 groups are encoded by syntenic orthologs that are colocalized to the same approximately 100–200 kilobase pair (Kbp) region in each species (Figure 1D). *PsAgo*6 and two *PsAgo*6 pseudogenes are also located in the *Ago*3, *Ago*4 and *Ago*5 region of *P. sojae* (Figure 1D, Table 2). The Ago1-5 proteins contain the conserved MID domain amino acids known to interact with small RNA guide strand 5′ phosphates [40], [41], and contain the conserved PIWI domain metal-ion-coordinating residues (aspartic acid, aspartic acid, histidine) that are required for endonucleolytic cleavage activity (slicing) [42], [43], [44], [45], [46] (Figure 1E). In contrast, *PsAgo*6 and *PrAgo*7 encode proteins that lack one or more conserved catalytic residues in the PIWI domain (Figure 1E), and could represent a class that functions through a mechanism other than slicing. Alternatively, *PsAgo*6 and *PrAgo*7 may be pseudogenes given that they have substitution per site rates that are higher than Ago proteins encoded by syntenic orthologs (Ago1-5) and similar to the putative product of the *PsAgo*5_ps pseudogene (Figure 1C). We conclude that the genomes of *P. infestans*, *P. sojae* and *P. ramorum* encode a highly conserved set of core RNA silencing components with two anciently diverged clades of *Dcl* and *Ago* genes.

**Table 2:** Putative *Ago* pseudogenes.

Locus	Parent	Locus accession	Position
*PsAgo*5_ps	*PsAgo*5	gm1.16110_g	Scaffold 7∶1941866–1945020+
*PsAgo*6_ps1	*PsAgo*6	fgenesh1_pg.4_#_281	Scaffold 4∶1597730–1599169−
*PsAgo*6_ps2	*PsAgo*6	gm1.10550_g	Scaffold 4∶1595135–1597461−
*PrAgo*1_ps1	*PrAgo*1	fgenesh1_pg.C_scaffold_1073000001	Scaffold 1073∶6488–7803−
*PrAgo*1_ps2	*PrAgo*1	fgenesh1_pg.C_scaffold_7000159	Scaffold 7∶706074–707446+
*PrAgo*3_ps	*PrAgo*3	gwEuk.107.27.1	Scaffold 107∶78418–78798+

### Phytophthora have Distinct Small RNA Populations

The *P. infestans*, *P. sojae* and *P. ramorum* genomes provide a platform to study small RNA biogenesis and function in *Phytophthora* as they span the genus [3], [9], [10]. In plants, animals, fungi and other eukaryotes, high-throughput sequencing of small RNA is a valuable experimental tool for understanding small RNA biology [47]. In particular, analysis of small RNA length, distribution across the genome and co-occurrence with annotated genomic features is useful for classifying small RNA into distinct pathways (for example, see [27], [48], [49], [50], [51], [52], [53], [54], [55]). To assess the small RNA landscape of *P. infestans*, *P. sojae* and *P. ramorum*, we prepared small RNA libraries for sequencing from mycelium tissue. Individually prepared small RNA libraries from *P. sojae* and *P. ramorum* mycelia were sequenced on the 454 pyrosequencing platform [56] and yielded 34,784 and 59,314 reads, respectively. Two biological replicate samples prepared from *P. infestans* mycelium were sequenced on the Illumina 1G sequencing platform [57] and yielded a total of 9,932,843 reads. Small RNA sequences were parsed from adaptors and reads from each library that aligned without mismatches to its respective genome were kept for further analysis. *P. sojae* had 22,226 aligned reads (16,609 unique sequences) with 140,022 genomic matches, *P. ramorum* had 31,821 aligned reads (25,304 unique sequences) with 160,953 genomic matches and *P. infestans* had 5,594,216 aligned reads (1,893,617 unique sequences) with 54,692,467 genomic matches. The differences in genomic matches per unique sequence between species are probably caused by differences in genome size [9], [10].

**Figure 2:**
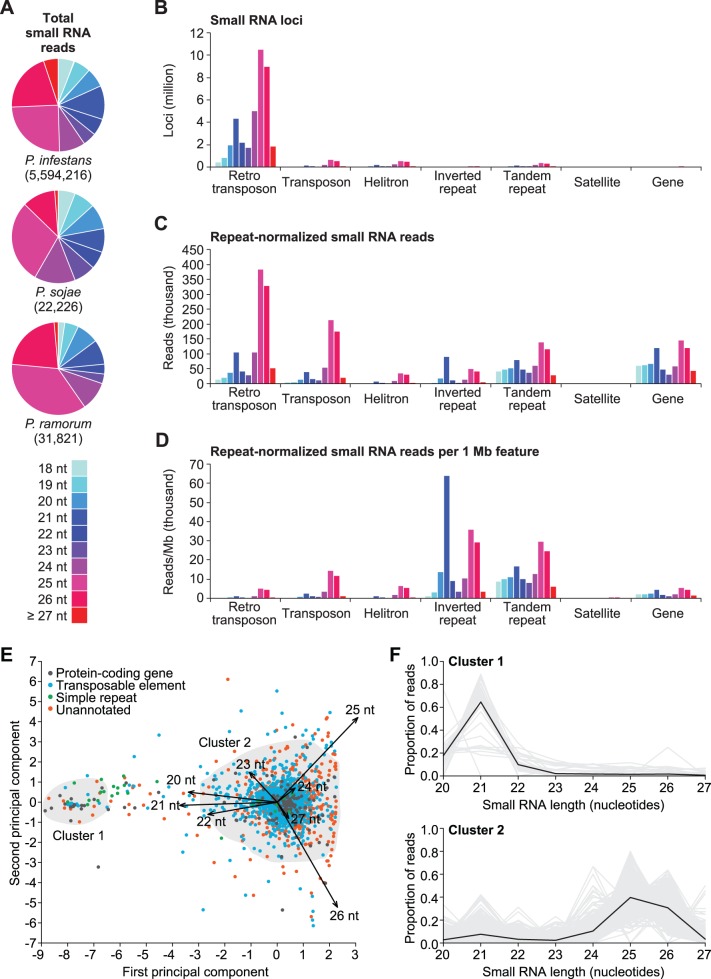
Classification of small RNA populations in three *Phytophthora* species. (A) Distribution of small RNA reads by size in *P. infestans*, *P. sojae* and *P. ramorum*. (B–D) Distribution of small RNA in *P. infestans* at retrotransposon, transposon, helitron transposon, inverted repeat, tandem repeat, satellite repeat and gene loci. Graphs are color-coded by small RNA size based on the legend in (A). (E) Biplot of principal components 1 and 2 from the PCA of *P. infestans* small RNA-generating segments. Small RNA-generating segments are color-coded based on overlapping features. Segments included in Cluster 1 or Cluster 2 from DBSCAN clustering of segments based on rotated small RNA size profile data are outlined. (F) Proportion of small RNA reads corresponding to 20-27-nucleotide small RNA for each small RNA-generating segment in DBSCAN Cluster 1 (upper) or Cluster 2 (lower). Gray lines are small RNA size profiles for individual segments and black lines are the cluster average profiles.

The genome-wide small RNA size profile for each of the three species was bimodal with peaks centered on 21- and 25-nucleotide RNAs (Figure 2). The most abundant small RNAs were 25 nucleotides long, but 26-nucleotide small RNAs were nearly as prevalent (Figure 2A). In total, 18-23-nucleotide and 24-30-nucleotide small RNA were 30–44% and 56–70% of the total aligned reads in each species, respectively. In *P. infestans*, the majority (84%) of small RNA genome alignments (loci) that could be assigned to an annotated feature category overlapped retrotransposons (Figure 2B). Small RNA read abundance was more distributed amongst *P. infestans* features, relative to small RNA loci, with 16–33% of reads overlapping transposons, tandem repeats, protein-coding genes and retrotransposons (16%, 19%, 22%, and 33%, respectively), and less than 10% overlapping inverted repeats, helitron transposons and satellite repeats (7%, 3% and <1%, respectively) (Figure 2C). The proportion of small RNA loci and reads that overlapped each *P. infestans* feature category was approximately proportional to the total genome space occupied by each feature type. Therefore, to better understand the relative small RNA-generating activity of each type of feature, we normalized the feature small RNA size profiles to account for total feature length. *P. infestans* inverted and tandem repeats had the highest small RNA-generating activity per megabase pair (Mbp) (Figure 2D). In contrast, *P. infestans* transposable elements and protein-coding genes had 4.6–11.4 times fewer reads per Mbp than inverted repeats (Figure 2D). Therefore, while transposable element and protein-coding gene loci are the most widespread sources of small RNA, inverted and tandem repeat loci may be the most potent triggers of small RNA biogenesis.

*P. infestans* feature-specific small RNA size profiles were similar to the genome-wide profile: bimodal with peaks at 21- and 25-nucleotide RNAs (Figure 2A–D). Retrotransposons, transposons, helitrons and satellite repeats had similar size profiles where 24-30-nucleotide small RNAs were 3.5–5.7 times more abundant than 18-23-nucleotide small RNAs. For tandem repeats, 24-30-nucleotide small RNAs were only 1.1 times more abundant than 18-23-nucleotide small RNAs. In contrast, *P. infestans* inverted repeats and protein-coding genes were the only feature types for which 18-23-nucleotide small RNAs were more abundant than 24-30-nucleotide small RNAs. Based on the whole-genome and feature-specific small RNA size profiles, we hypothesized that *Phytophthora* species produce two small RNA size classes. To explore this possibility, we analyzed the size distributions of small RNA-generating loci using principal component analysis (PCA). Small RNA generating-loci were defined as segments of the *P. infestans* genome that contained mapped reads with up to 50 nucleotides of space between consecutive small RNAs. Segments were kept for further analysis if they contained at least one distinct pair of small RNAs that overlapped on opposite strands with 2-nucleotide 3′ overhangs, as expected for Dicer products [11]. We reasoned that segments that contained putative Dicer-duplexes were more likely to be *bona fide* small RNA-generating loci. For each of the 2,328 *P. infestans* segments that contained a Dicer-duplex pair (2,812,472 total reads), a proportional small RNA size profile was constructed, to remove abundance differences between segments, by calculating the proportion of small RNA reads for each size (20–27 nucleotides) relative to the total segment reads (Table A in File S1). PCA was done on the segments using a model that treated the proportion for each small RNA size (20–27 nucleotides) as an independent variable. The first principal component explained 30% of the variance and differentiated segments into two groups based on predominant small RNA sizes: 20-23-nucleotides versus 24-27-nucleotides (Figure 2E). The second principal component explained 15% of the variance and primarily differentiated segments based on the proportion of 25- and 26-nucleotide small RNA (Figure 2E). To further validate the size class differentiation, segments were clustered on the rotated data from the first and second principal components using the DBSCAN (density-based spatial clustering of applications with noise) algorithm [58]. DBSCAN clustering produced two clusters (Figure 2E, Cluster 1 and Cluster 2). Cluster 1 contained 52 segments that produced primarily 21-nucleotide small RNAs (Figure 2F). Cluster 2 contained 2,141 segments that produced primarily 25/26-nucleotide small RNAs (Figure 2F). Clustering did not result in a noticeable differentiation between segments with different proportions of 25- and 26-nucleotide small RNA (Figure 2E). The PCA and clustering results are consistent with our hypothesis that *Phytophthora* species produce two primary small RNA sizes centered around 21- and 25-nucleotides.

Taken together, the size distributions, PCA and clustering analyses are all consistent with the presence of two size classes of *Phytophthora* small RNA. The presence of two small RNA size classes and two *Dcl* orthologs in each species raises the possibility that each *Dcl* produces RNAs of one of the two size groups, similar to the way that plants partition small RNA pathways with specialized DCL proteins [59]. Consistent with this model, RNAi knockdown of *PiDcl*1 was recently shown to disrupt accumulation of 21-, but not 25/26-nucleotide small RNAs [28]. Additionally, *Phytophthora* Ago proteins may be partitioned into two or more functional groups that might stabilize 21- or 25/26-nucleotide small RNAs. Further analysis of RNA silencing-deficient lines will be required to confirm this hypothesis and identify any functional differences between the two small RNA size classes.

### Genome-wide Distribution of Small RNA-generating Loci

**Figure 3:**
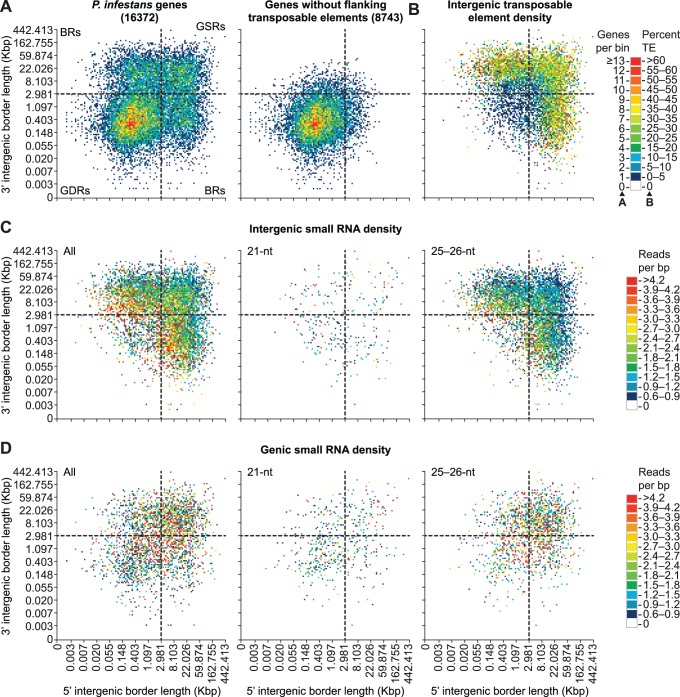
Distribution of small RNA in the *P. infestans* genome. (A–D) Two-dimensional binning of *P. infestans* genes based on the length of 5′ and 3′ flanking intergenic regions. Plots are divided into quadrants with dashed lines: gene-dense regions (GDRs), gene-sparse regions (GSRs), and border regions (BRs). (A) Heatmap color scale represents the number of genes per bin for all genes (left) and genes without transposable elements in flanking intergenic regions (Data from [9]). (B) Genes were binned as in (A), but the heatmap color scale represents the percentage of base pairs in both the 5′ and 3′ flanking intergenic regions that were occupied by transposable elements. (C and D) Genes were binned as in (A), but the heatmap color scale represents the repeat-normalized small RNA reads per base pair in either both the 5′ and 3′ flanking intergenic regions (C) or the gene body (D).

The feature types in Figure 2, other than inverted repeats, were associated with abundant 25/26-nucleotide small RNAs, relative to 21-nucleotide small RNAs. Furthermore, the relative proportion of feature types in Cluster 1 and 2 (21- versus 25/26-nucleotide small RNA-generating segments, Figure 2E) were significantly different (p<0.001, Fisher’s exact test). Protein-coding gene and simple repeat segments were 7.0 (95% confidence interval: 3.5–13.4) and 36.5 (95% confidence interval: 11.0–116.1) times more likely to generate predominantly 21-nucleotide small RNAs, respectively (p<0.001, Fisher’s exact test). In contrast, transposable element segments were 3.2 (95% confidence interval: 1.8–6.0) times more likely to generate predominantly 25/26-nucleotide small RNAs (p<0.001, Fisher’s exact test). The *P. infestans* genome (240 Mbp) is more than twice as large as the *P. sojae* and *P. ramorum* genomes (95 and 65 Mbp, respectively) due to recent proliferation of transposable elements [9], [10]. Expansion of the *P. infestans* genome was not uniform and resulted in a distinct genome architecture that consists of blocks of gene-dense and gene-sparse regions [9]. Genome-wide differences in gene density can be visualized by binning genes into a two-dimensional matrix based on the length of 5′ and 3′ intergenic border regions [9]. As shown previously [9], most genes in *P. infestans* are flanked by intergenic regions between 20 bp and 3 Kbp long (gene-dense regions, GDRs), while another set are flanked by at least one intergenic region that is 5 Kbp or larger (gene-sparse regions, GSRs and border regions, BRs) (Figure 3A, left panel). GDRs and GSRs are markedly different; genes that lack transposable elements in their flanking intergenic regions are primarily in GDRs (Figure 3A, right panel). Similarly, the proportion of intergenic space occupied by transposable elements was lowest in GDRs (median = 0%) and highest in GSRs and BRs (median = 22%) (Figure 3B). A similar pattern was observed for intergenic small RNAs. Intergenic segments from gene-sparse, transposon-rich regions had the highest density (reads per bp) of all small RNAs, and in particular, 25/26-nucleotide small RNAs (Figure 3C). In contrast, intergenic segments generally had low 21-nucleotide small RNA density and were distributed between both GDRs and GSRs (Figure 3C). Small RNA density within genes was more variable, but was generally highest for 25/26-nucleotide small RNAs (Figure 3D). Unlike intergenic small RNAs, intragenic 25/26-nucleotide small RNA density was more pronounced in genes from GSRs than BRs (Figure 3D). Genes generally had low 21-nucleotide small RNA density (Figure 3D), with the exception of a few gene families discussed below.

The distribution of 25/26-nucleotide small RNAs across the *P. infestans* genome coincides with the location of transposable elements and low gene density. Additionally, we observed enrichment of transposable element loci at 25/26-nucleotide-generating segments of the *P. infestans* genome. Therefore, we hypothesize that 25/26-nucleotide small RNAs function to repress transposable elements in *Phytophthora* species. In agreement with our observations, *P. sojae* strains that contain naturally silenced alleles of the avirulence gene *Avr*3a were shown to have abundant 24-26-nucleotide small RNAs at the *Avr*3a locus [60]. Furthermore, heritable transgenerational silencing of *Avr*3a was demonstrated in crosses between silenced and non-silenced strains, suggesting an epigenetic mode of inheritance [60]. In other RNA silencing systems, transposable elements and other repetitive DNA are silenced through heterochromatin modifications [61]. In plants, 24-nucleotide small RNAs guide AGO proteins to target loci, resulting in DNA methylation and ultimately repressive chromatin modifications [22]. The mechanism by which 25/26-nucleotide small RNAs might silence targets is unknown, but previous work suggests that transcriptional silencing in *Phytophthora* is mediated through chromatin modifications and not DNA methylation [62], [63].

### Distinct Small RNA Populations from Protein-coding Gene and Pseudogene Loci

**Figure 4:**
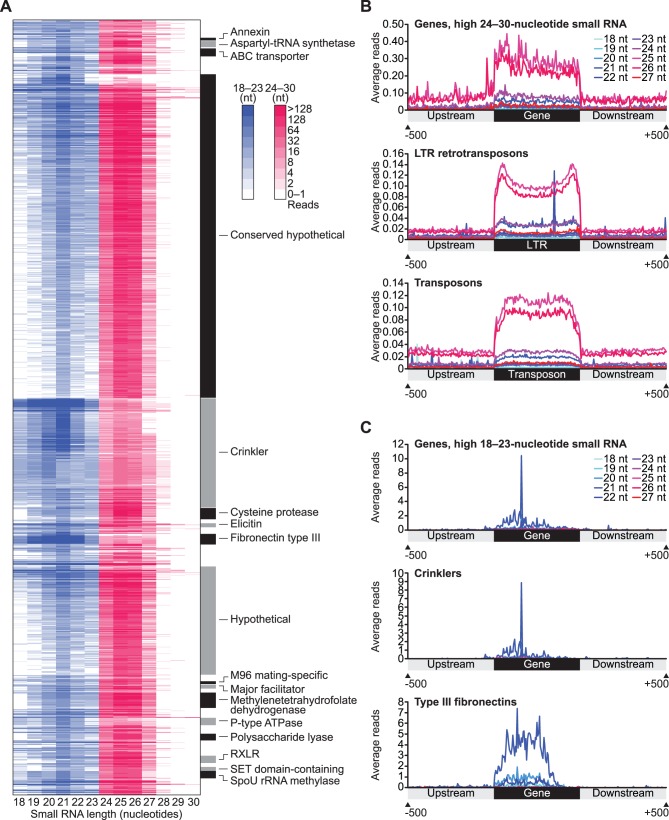
Small RNA from protein-coding gene and pseudogene loci in *P. infestans*. (A) Heatmap-based size profile of 18-30-nucleotide small RNA reads mapping to 1,166 *P. infestans* genes that overlapped at least 100 total small RNA reads. Gene annotation groups with at least four genes are labeled with alternating black and gray boxes. (B and C) Regional metaplots with average 18-27-nucleotide small RNA reads per position. X-axis positions are relative scale (0–100%) for each region. Genes from (A) with more 24-30- than 18-23-nucleotide small RNA reads, LTR retrotransposons and transposons are shown (B). Genes from (A) with more 18-23- than 24-30-nucleotide small RNA reads, crinklers and type III fibronectins are shown (C).

As described above, the majority of small RNAs in *P. infestans* are derived from transposable elements, repeats and other intergenic regions, but some genes were identified at small RNA-generating loci (Figure 2, Figure 3). To examine small RNA-generating genic loci in more detail, we identified 1,166 *P. infestans* genes and pseudogenes that overlapped at least 100 total small RNA reads. Gene annotations were updated using BLAST2GO [64], and genes were grouped by annotation and small RNA size profile (Figure 4A, Table B in File S1). Of the 1,166 genes, 875 genes (including 17 pseudogenes) overlapped predominantly 24-30-nucleotide small RNAs and 291 genes (including 54 pseudogenes) overlapped predominantly 18-23-nucleotide small RNAs (Figure 4A, Table B in File S1). The majority (55.8%) of the small RNA-generating genes were of unknown type (hypothetical and conserved hypothetical), and 90% of the genes in these categories were associated primarily with 24-30-nucleotide small RNAs (Figure 4A, Table B in File S1). Annotated families with four or more members in the small RNA-generating set included genes encoding 4 annexins (ANX), 9 aspartyl-tRNA synthetases (AARS), 12 ATP-binding cassette (ABC) transporters, 163 crinkler effectors (CRN) (including 68 pseudogenes), 17 C48 family cysteine proteases (C48), 6 elicitins (ELI) (including 2 pseudogenes), 16 type III fibronectins (FN3), 4 M96 mating-specific proteins (M96) (including 1 pseudogene), 6 major facilitator superfamily (MFS) transporters, 23 methylenetetrahydrofolate dehydrogenases (MTHFD), 11 P-type ATPases (P-ATPase), 10 polysaccharide lyases (PL), 11 RXLR (Arg-X-Leu-Arg, where X is any amino acid) effectors, 6 SET domain-containing proteins and 11 SpoU rRNA methyltransferases (Figure 4A, Table B in File S1). The small RNAs from *AARS*, *C48*, *M96*, *MTHFD*, *PL*, *RXLR*, *SET* and *SpoU* genes were predominantly 24-30-nucleotides long (60–85% of the total reads on average per family). Interestingly, five of the eight families (*C48, PL, RXLR, SET* and *SpoU*) are enriched in gene-sparse regions [65]. Given that 24-30-nucleotide small RNAs were enriched at transposable element loci, or in gene-sparse, transposable element-rich regions (Figure 2, Figure 3), genes that overlap 24-30-nucleotide small RNA-generating regions may represent loci that were captured by transposable element silencing machinery either in cis or in trans. The distribution of small RNAs within, upstream, and downstream of genes that overlapped predominantly 24-30-nucleotide small RNAs was similar to transposons and LTR retrotransposons where 25/26-nucletide small RNA abundance was greatest within genes or transposable elements and continued, at a lower level, into the 500 bp upstream and downstream regions (Figure 4B). The higher 25/26-nucleotide small RNA abundance within genes and transposable elements, relative to flanking regions suggests that silencing is generally feature-specific. The appreciable 25/26-nucleotide small RNA density in the upstream and downstream flanking regions may arise from local spreading of silencing.

**Figure 5:**
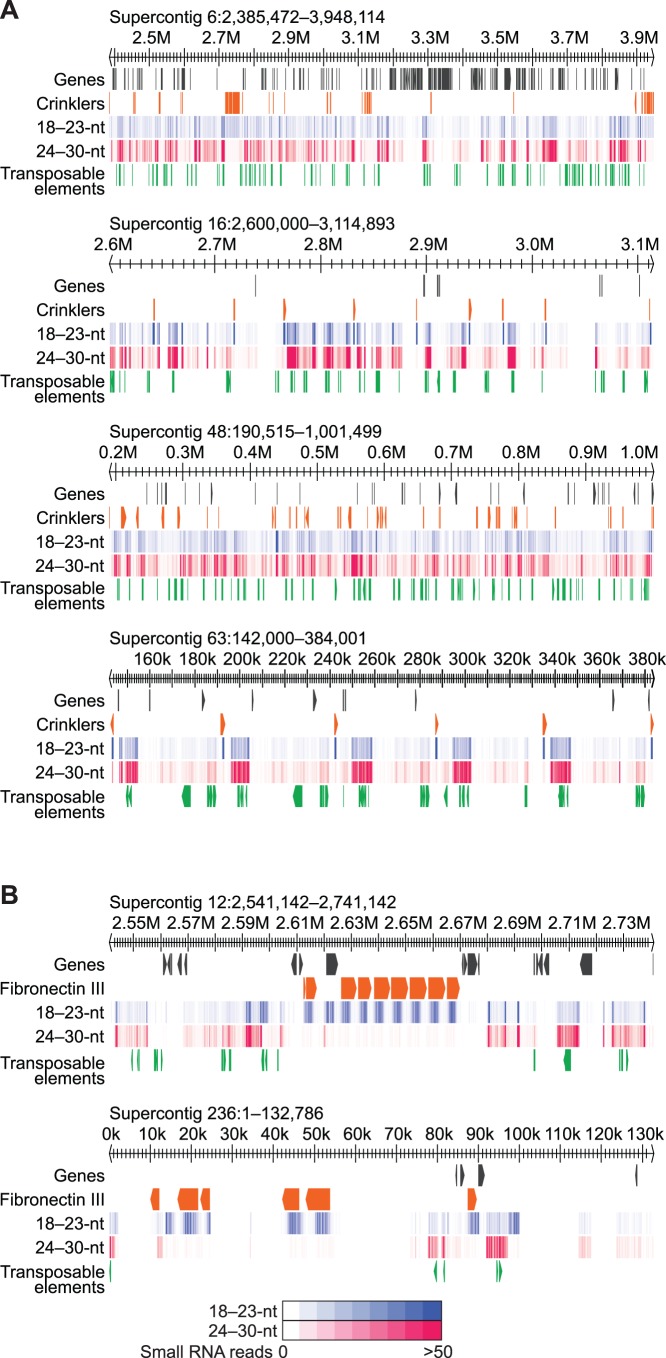
Regions of *P. infestans* containing clusters of *CRN* and *FN3* genes and pseudogenes. (A and B) Genomic regions containing *CRN* and *FN3* genes. The position of genes, transposable elements, *CRN* genes (A) and *FN3* genes (B) are shown. 18-23- and 24-30-nucleotide small RNA read density heatmaps are shown (see legend at the bottom of B).

Unlike most other features in the *P. infestans* genome, some genes encoding ANX, CRN, FN3 and P-ATPases overlapped small RNAs that were primarily 18-23-nucleotides long (64–95% of the total reads on average per family). In contrast to genes associated with 25/26-nucleotide small RNAs, 18-23-nucleotide small RNAs, and in particular 21-nucleotide small RNAs, at *ANX*, *CRN*, *FN3* and *P-ATPase* loci were more abundant on average and were almost exclusively located in the gene body (Figure 4B and C). In particular, *CRN* and *FN3* genes produced large clusters of abundant 21-nucleotide small RNAs (Figure 4A, C). In the *P. infestans* genome, *CRN* genes and pseudogenes are found in several large gene clusters and are often grouped with *CRN* genes with the same domain architecture [9]. Of the 163 *CRN* genes and pseudogenes, 135 genes were associated with predominantly 18-23-nucleotide small RNAs, and 123 of these *CRN* genes were from or were found clustered with *CRN* with one of four domain architectures (Figure 5A). *CRN* groups that generate 21-nucleotide small RNAs were the DN5-type that were found primarily (14 of 17) on Supercontig 16 and 63, the DC-type that were found primarily (28 of 34) on Supercontig 6, and the D2 and DXZ types that were found together primarily (63 of 73) on Supercontig 31, 48, 82, 85, 69, 104 and 143 (Figure 5A). Interestingly, *FN3* genes that produced 21-nucleotide small RNAs were also found in dense gene clusters (Figure 5B). It is unclear what mechanism generates 21- rather than 25/26-nucleotide small RNAs from *ANX*, *CRN*, *FN3* and *P-ATPase* genes. Because inverted repeats (Figure 2) and miRNA (see below) are associated with 18-23-nucleotide small RNAs, one possibility is that 21-nucleotide small RNAs from *ANX*, *CRN*, *FN3* and *P-ATPase* are generated through an Rdr-independent mechanism that involves intramolecular double-stranded RNA formation. Small RNAs produced from a folded single-stranded RNA precursor are expected to match only the transcribed precursor strand, but 95% of genes that were associated with predominantly 18-23-nucleotide small RNAs had at least one antisense read, suggesting that these precursors are converted to double-stranded RNA through Rdr activity.

### A Conserved MIRNA Family in Phytophthora

**Figure 6:**
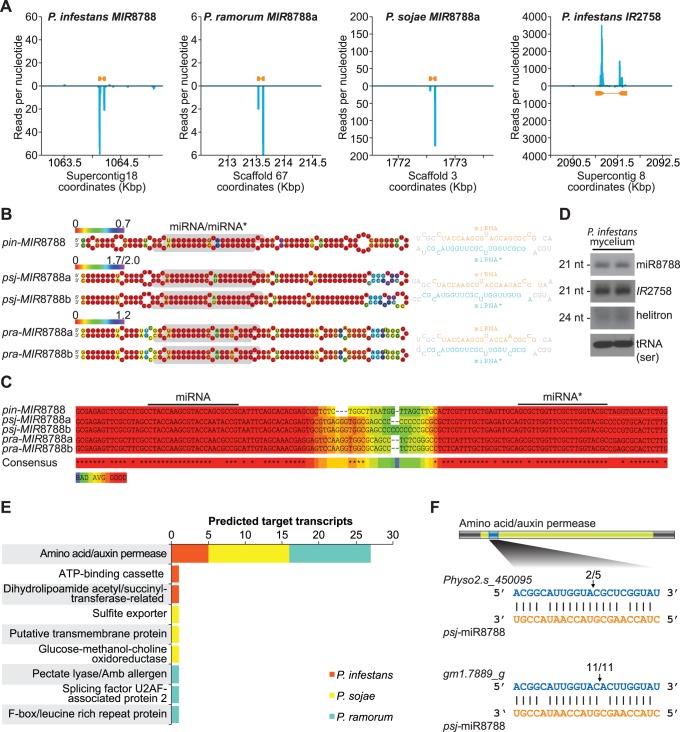
Computational and molecular characterization of *Phytophthora MIRNA* genes. (A) Single-nucleotide resolution small RNA read density in regions containing *pin-MIR*8788, *pra-MIR*8788a, *psj-MIR*8788a and *pin-IR*2758. (B) *MIR*8788 foldback structures predicted with RNAfold [90]. Ribonucleotides are colored by base-pairing entropy. (C) T-coffee alignment of DNA sequences corresponding to the *MIR*8788 foldbacks. Alignment quality is shown as a heatmap. (D) Northern blot detection of the *pin*-miR8788 guide RNA and *pin-IR*2758- and *Pi*-helitron-derived small RNA in *P. infestans* mycelia tissue. Northern blot detection of serine-tRNA was included as a loading standard. (E) Summary of TargetFinder results for predicted target transcripts of miR8788. (F) RLM-5′RACE validation of *psj*-miR8788-guided cleavage of *AAAP* transcripts. Arrows indicate the detected cleavage position with the number of clones supporting cleavage out of the total tested. The representative structure of *AAAP* transcripts is shown with the AAAP domain and miRNA target site highlighted.

To address the question of whether oomycetes have precisely-processed miRNAs similar to those in other eukaryotes, we designed a pipeline to identify candidate *MIRNA* genes using high-throughput small RNA sequencing data from *P. infestans*, *P. sojae* and *P. ramorum*. First, small RNA that mapped to transposable element or structural RNA loci were removed from further analysis. Second, 20-22-nucleotide small RNA sequences were filtered to limit further analysis to sequences with two or more reads and ten or fewer matches to the genome. The remaining sequences and surrounding genomic regions were extracted and RNA structural analysis was done to identify foldback structures with *MIRNA*-like characteristics. Overlapping loci were consolidated and filtered for strand bias (at least 95% of the small RNA reads were required to originate from one strand) to eliminate Rdr-dependent small RNA-generating regions. Finally, for each remaining foldback, the most abundant sequence was considered the mature miRNA and was required to have a complementary sequence with two-nucleotide 3′ overhangs (miRNA*) from the opposite arm of the foldback. Two loci each from *P. infestans, P. sojae* and *P. ramorum* matched our criteria (Figure 6A). One locus from *P. infestans* was a 714-nucleotide inverted repeat (*pin-IR*2758) that produced heterogeneous 21-nucleotide siRNA and was therefore not considered a *MIRNA* (Figure 6A). The two putative *MIRNA* loci in *P. ramorum* and *P. sojae* were from the same family (*MIR*8788) and were related to one of the loci from *P. infestans*. The foldback structures of all *MIR*8788 homologs were strikingly similar, and multiple sequence alignment of the primary foldback sequences also showed that the foldbacks were highly conserved (Figure 6B and C). *MIR*8788 foldback arms were particularly conserved, relative to loop sequences, possibly due to functional sequences present in the foldback stem (Figure 6C). Additionally, the first 15 nucleotides of the miRNA guide strand, which corresponds to the putative miRNA-target RNA seed-pairing region, were preserved in all *MIR*8788 foldbacks (Figure 6C). The miRNA and miRNA* 5′ end processing positions within the *MIR*8788 foldbacks (deduced from the sequenced small RNAs) were consistent while the 3′ ends were variable (Figure S1). Positioning of the miRNA and miRNA* 5′ ends suggests that Dcl processing of *MIR*8788 in *P. infestans* and *P. ramorum* results in a 21-nucleotide guide strand, but results in a 22-nucleotide guide in *P. sojae* (Figure S1). We confirmed the presence and size of *pin*-miR8788 by northern blot assay (Figure 6D). In plants, miRNA are protected from 3′–>5′ degradation by 2′-O-methyl modification of the 3′ terminal nucleotide by the methyltransferase HUA ENHANCER1 (HEN1) [66]. The 3′ end heterogeneity we observed might result from trimming of the small RNAs after Dcl processing because *P. infestans* small RNAs were found to have unmodified 3′ terminal nucleotides, and *P. infestans* lacks a clear HEN1 homolog [28].

We used TargetFinder [67] to computationally predict target transcripts for miR8788 in *P. sojae*, *P. ramorum* and *P. infestans*. The best-scoring target RNAs for all three species were from the amino acid/auxin permease family (AAAP) (Figure 6E). In addition, *pin*-miR8788, *psj*-miR8788 and *pra*-miR8788 were predicted to target 5, 11 and 11 *AAAP* transcripts, respectively, while predicted targeting of single genes from other families was limited to specific species (Figure 6E). We used RNA ligase-mediated rapid amplification of 5′ complementary DNA ends (RLM-5′RACE) to assess two *AAAP* transcripts for miR8788-guided cleavage in *P. sojae*. *Physo2.s_450095* cleavage products with cleavage between the canonical positions 10 and 11 relative to the 5′ end of *psj*-miR8788 were detected (Figure 6F). *Gm1.7889_g* cleavage products were also detected within the *psj*-miR8788 target site, but cleavage was detected between positions 9 and 10 (Figure 6F). Further work would be required to assign biological significance to regulation of *AAAP* transcripts by *MIR*8788.

Evidence that *MIR*8788 is a *bona fide* miRNA-generating locus is strong, but it is perhaps surprising that only one *MIRNA* family was found in our analysis. To assess possible biases in our computational pipeline, we independently analyzed the *P. infestans* small RNA libraries using the ShortStack pipeline [68]. Results from ShortStack matched those from our pipeline in predicting only a single *MIRNA* locus, *MIR*8788. These analyses may have missed miRNAs that were not represented in our libraries because of specific spatiotemporal or environmental-dependent patterns. Furthermore, our analysis relied on libraries from mycelium grown in broth and it is possible that additional *MIRNA* loci specific to certain life-stages, particularly during host-pathogen interaction, remain to be discovered. Alternatively, the concept of a distinct miRNA biogenesis pathway may not be relevant to *Phytophthora* species if their small RNA pathways produce ∼21-nucleotide miRNA-like sequences from hairpin RNAs with differing precision and processing efficiencies, producing sets of small RNAs that range from heterogeneous (*pin-IR*2758) to precise (*MIR*8788) (Figure 6A). Indeed, the *P. infestans*, *P. ramorum* and *P. sojae* genomes all contained inverted repeats predicted to produce hairpin RNAs that are processed into ∼21-nucleotide small RNAs (Figure 2B–D, Table C in File S1). It may be that these *MIRNA*-like inverted repeats have functions similar to plant and animal *MIRNA* genes. If this is the case, computational prediction of these loci and their targets will need to be refined, particularly because of their relatively imprecise processing by Dcls.

## Conclusions

In plants, animals and fungi, duplication and diversification of genes that encode DCR/DCL, RDR, AGO and other accessory proteins has resulted in the evolution of distinct RNA silencing pathways [24]. We find that *Phytophthora* species have a conserved set of core RNA silencing machinery, including one Rdr, two Dcl and four Ago proteins, with some species-specific duplicates. Our detailed analysis of small RNA sizes and distribution across the *P. infestans* genome, as well as small RNA sizes from *P. sojae* and *P. ramorum*, supports the presence of two primary, distinct small RNA size classes: 21- and 25/26-nucleotides. The 21- and 25/26-nucleotide small RNAs are not just distinct in size but are also generated from distinct features, with 25/26-nucleotide small RNAs predominantly associated with transposable elements and 21-nucleotide small RNAs associated with inverted repeats and several gene families. These results are largely in agreement with Vetukuri et al. [28] except that we do not provide evidence for or against a 32-nucleotide size class, as these RNAs were not present in our data sets. Taken together, we propose that *Phytophthora* have at least two distinct small RNA pathways that may be partitioned by Rdr, Dcl and Ago diversification as well as small RNA size. In this model, we propose that 25/26-nucleotide small RNAs define a siRNA pathway that targets transposable elements and other repetitive features and silences target loci through an epigenetic mechanism. Besides a role for genome defense and evolution of genome structure, epigenetic regulation of pathogenicity factors has important implications for the evolution of isolates and host range [60].

Additionally, we propose that 21-nucleotide small RNAs define at least a second pathway that regulates targets by post-transcriptional control. The presence of a second pathway for small RNA biogenesis is further supported by the observed ancient divergence of Dcl1 and Dcl2, and the deep split in the Ago tree separating Ago1/2 from the other Agos, reflecting independent evolution. Some 21-nucleotide-generating loci, such as *CRN* and *FN3*, appear to be siRNA and likely are dependent on Rdr activity. In contrast, other 21-nucleotide small RNAs may be generated from hairpin RNA (e.g. *MIR*8788) without the need for Rdr activity. It is unclear what the function of 21-nucleotide small RNAs is, but given the large number of *P. infestans CRN* genes associated with 21-nucleotide small RNAs they may be important components of pathogenicity, host range or variability of effector repertoire between isolates.

## Materials and Methods

### Phytophthora Strains and Growth Conditions

Mycelium was grown from the sequenced strains of three *Phytophthora* species; *Phytophthora ramorum* Pr-102, *Phytophthora sojae* P6497 and *Phytophthora infestans* T30-4 [9], [10]. *Phytophthora* isolates were maintained on cleared 10% V8 agar medium (100 mL V8 juice; 1 g CaCO_3_; 30 mg/L β-sitosterol (EMD Chemicals, Incorporated); 15 g agar; 900 mL deionized water) in a 20°C incubator in the dark [69]. Mycelia were grown in V8 broth (V8 agar medium without agar) on a rotating shaker in flasks and collected after 7 days for *P. sojae* and *P. ramorum* and after 4 days for *P. infestans*.

### Total RNA Preparation

Total RNA was isolated as described previously [49]. Briefly, mycelium samples were frozen in liquid nitrogen, ground into a fine powder and homogenized in Trizol (1 g∶10 mL, Life Technologies). After adding 2 mL chloroform per 10 mL Trizol, samples were mixed, incubated at room temperature for 5 minutes and centrifuged at 8400×g for 10 minutes. Two additional chloroform extractions were done before RNA was precipitated in 0.7 volumes isopropanol for 20 minutes at room temperature and spun for 30 minutes at 8400×g. Minimally dried pellets were resuspended in 200 µL 0.1X TE, extracted 2 times with phenol:chloroform:isoamyl alcohol (50∶49∶1), and once with chloroform. Total RNA was precipitated with 5 M ammonium acetate and ethanol at −80°C overnight, spun at 12000×g for 30 minutes at 4°C, resuspended in 100 microliters 0.1X TE and quantitated by Nanodrop (Thermo Scientific).

### Small RNA Library Construction and Sequencing

Small RNA libraries for *P. ramorum* and *P. sojae* were prepared for sequencing on the 454 platform using 5′ adaptors 5′-ATCGTAG**GCUG**CUGAUA-3′ and 5′-ATCGTAG**GCCA**CUGAUA-3′ for *P. sojae* and *P. ramorum*, respectively, with strain-specific bases bolded, as described previously [49]. *P. infestans* libraries were prepared for sequencing on the Illumina 1 G platform as described previously [70] except that no RNA spike-in standards were added and no barcoded adaptors were used.

### Small RNA Data Processing

Sequencing reads were parsed to remove adaptors and collapsed to a unique set with read counts. Parsed sequences from each species were aligned to their respective genome using BOWTIE v0.12.8 [71] with settings that limit the results to perfect matches only (–f –v 0–a –S). Aligned small RNAs were stored in Sequence Alignment/Map (SAM) format and were accessed using SAMTOOLS [72]. The annotated genomes for *P. sojae* (v3.0) and *P. ramorum* (v1.1) were obtained from the Joint Genome Institute (JGI) [6]. The annotated genome for *P. infestans* was provided by the Broad Institute [9]. Where noted, reads were repeat-normalized by dividing the read counts by the total number of locations in the genome the small RNA mapped to.

### Identification of Rdr, Dcl and Ago Homologs

Putative homologs of the small RNA biogenesis and effector genes *Rdr*, *Dcl*, and *Ago* were located using tBLASTn [73]. Plant and animal homolog protein sequences were used as the query against both transcript and genome databases from *P. sojae*, *P. ramorum*, and *P. infestans* [9], [10]. For *PiDcl*2, MEGABLAST [73] was used to align *PsDcl2* to the *P. infestans* whole-genome shotgun reads at the NCBI Trace Archive (File S2). CAP3 [74] was used to assembly the whole-genome shotgun reads into a 3962 bp contig (File S2). In cases where BLAST results from transcript and genome databases overlapped at the same locus, the annotated gene model was used and is reported in Table 1. *PiDcl*2 and genomic BLAST results for *PrRdr*1, *PrAgo*3 and *PrAgo*7 did not have corresponding gene models so GENSCAN [75] and FGENESH [76] were used to predict gene structures (File S2). Conserved domains were annotated using the PFAM database [77] for the complete set of proteins identified. Proteins were filtered based on the presence of conserved domain architecture specific to the gene families: Rdr proteins were required to contain an RNA-dependent RNA polymerase domain [78], Ago proteins were required to contain a DUF1785, PAZ and PIWI domain [79], and Dcl proteins were required to contain two RNaseIII domains [80]. Six *Ago* genes had one or more missing or truncated domain and were considered pseudogenes (Table 2). Gene names were assigned to orthologs and paralogs based on phylogenetic and syntenic analysis (see below) and to preserve previously published nomenclature [28], [30] (Table 1).

### Phylogenetic Analysis

Phylogenetic analysis was done using the amino acid sequences of the RdRP domain, RNaseIII-RNaseIII domains region and DUF1785-PAZ-PIWI domains region. PsAgo6_ps1, PsAgo6_ps2, PrAgo1_ps1, PrAgo1_ps2 and PrAgo3_ps were not included in the Ago phylogenetic analysis because each had missing or truncated DUF1785, PAZ or PIWI domains. For each family, protein multiple sequence alignments were built with MAFFT [81]. The aligned sequences were imported into Molecular Evolutionary Genetics Analysis (MEGA v5.05) [82] and trimmed to exclude positions that contained a gap in any sequence. Maximum Likelihood inference with RAxML v7.3.0 [83] was used with a GAMMA model of rate heterogeneity and the LG substitution matrix to build phylogenetic trees using the majority-rule consensus criterion. Support for clades was obtained using 1,000 bootstraps. *Arabidopsis thaliana* RDR and DCL proteins were included as outgroups for the Rdr and Dcl analyses, respectively, and *T. gondii* Ago1 was included as the outgroup for the Ago analysis. Consensus trees were drawn with DENDROSCOPE 3.2.4 [84]. Synteny analysis of *Ago*1-5 genes was done using the JGI Genome Portal and VISTA Point browser [85], [86].

### Statistical Analyses

All statistical tests were done using R v2.15 [87]. Principal component analysis was done by the scalar value decomposition method on centered and scaled data with the prcomp function (stats package). For PCA, the size profiles for *P. infestans* small RNA-generating segments were normalized to remove abundance differences between segments but maintain the relative contribution of each size class to the overall segment profile. For each segment, the relative contribution of each small RNA size class between 20- and 27-nucleotides was calculated by dividing the total size class reads by the total reads for the segment. DBSCAN clustering [58] was done with the dbscan function (fpc package [88]) using a minimum cluster size of 30 and a maximum distance (eps) of 0.8. Fisher’s exact test was done with the fisher.test function (stats package).

### Identification and Analysis of MIRNA Genes

A computational pipeline was used to identify putative miRNA from among the *Phytophthora* small RNA datasets. First, small RNA arising from transposable elements and structural RNA loci were removed. Second, only 20-22-nucleotide small RNAs with two or more reads and ten or fewer matches to the genome were considered. Third, an initial foldback assessment was done with small RNA-flanking sequence (1 Kbp) using EINVERTED (-gap 12 -threshold 10 -match 3 -mismatch -4) [89]. Small RNAs located within EINVERTED alignment results were analyzed further, and overlapping loci were consolidated. Fourth, secondary structure analysis was done using RNAfold [90] on consolidated loci where at least 95% of the small RNA reads were from one strand. The most abundant small RNA from each locus had to be located in a stem region and was considered a miRNA if a small RNA matching the predicted miRNA* was sequenced in our datasets.

To assess possible biases in our computational pipeline, we independently analyzed the *P. infestans* small RNA libraries using the ShortStack pipeline v0.4 [68]. Aligned small RNA reads in SAM format were compressed, sorted and indexed in BAM format using SAMTOOLS. Prep_bam.pl v0.1.1 from ShortStack was used to prepare the BAM file for the ShortStack pipeline. ShortStack was run with default settings except that the small RNA size range was set to 18–26 nucleotides (–dicermin 18–dicermax 26).

### miRNA Target Prediction

Targets for miR8788 were predicted for *P. sojae*, *P. ramorum* and *P. infestans* transcripts using TargetFinder v1.6 (http://carringtonlab.org/resources/targetfinder) [67] with a score cutoff of 5 (-c 5).

### Northern Blot Analysis

Total RNA (10 µg) from *P. infestans* mycelium tissue was resolved by denaturing 17% polyacrylamide-urea gel electrophoresis. For size standards, 21- and 24-nucleotide synthetic RNA markers (5′-pUGUGGCCGAGGAUGUUUCCGU-3′ and 5′-pUUGUGGCCGAGGAUGUUUCCGUCC-3′, respectively) were also resolved. RNA was transferred to a positively charged nylon membrane (Nytran SuPerCharge, Whatman) by semi-dry electrotransfer. RNA was UV-crosslinked to the membrane twice at 1200 Joules. DNA probes for *pin*-miR8788 (5′-GCGGCGCTGGTACGCTTGGTAG-3′) and serine-tRNA (5′-CTGTGAGATTCGAACTCACGC-3′) were end-labeled by phosphorylation with γ^32^P-ATP and Optikinase (Amersham). Membranes were incubated with hybridization solution (PerfectHyb Plus, Sigma) at 38°C for 1 hour and then incubated with probes at 38°C overnight.

### RLM-5′RACE

The *P. sojae* candidate *psj-*miR8788 target transcripts *Physo2.s_450095* and *gm1.7889_g* were analyzed for miRNA-guided cleavage by modified RLM-5′RACE using the GeneRacer kit (Life Technologies) as described [91]. RT-PCR was done using nested 5′ linker primers and gene-specific primers located downstream of the predicted *psj*-miR8788 binding site. Gene-specific primers were 5′-AGCAGCGAGCCGCCCAGCACGAGGAA-3′ and 5′-TGGGCACCAGGCACACGGGCAGCAC-3′ for *Physo2.s_450095* and 5′-AGCGTGGGAATGAGGCACACCGGCA-3′ and 5′-AGGTCGGGGAACGGCACACTCGGGT-3′ for *gm1.7889_g.* PCR products were cloned and sequenced to detect RNA fragment 5′ ends.

### Accessions

*P. tetraurelia* sequences were extracted from ParameciumDB [92], [93]: Rdr1 [GSPATP00024768001]; Rdr2 [GSPATP00036857001]; Dcl1 [GSPATP00021751001]; Dcl2a [GSPATP00027456001]; Dcl2b [GSPATP00032258001]. *T. thermophilia* sequences were extracted from the *Tetrahymena* Genome Database [94]: Rdr1 [TTHERM_00853150]; Dcl1 [TTHERM_00284230]; Dcl2 [TTHERM_00316190]. *T. gondii* sequences were extracted from the NCBI protein database: Rdr1 [XP_002371017]; Ago1 [XP_002364262]. *A. thaliana* sequences were extracted from The Arabidopsis Information Resource (TAIR v10) [95]: RDR1 [AT1G14790]; RDR2 [AT4G11130]; RDR6 [AT3G49500]; DCL1 [AT1G01040]: DCL2 [AT3G03300]; DCL3 [AT3G43920]; DCL4 [AT5G20320]; AGO1 [AT1G48410]. *Homo sapiens* AGO2 [Q9UKV8] and *Drosophila melanogaster* AGO1 [Q27IR0] were extracted from UniProtKB [96]. Small RNA datasets were uploaded to the Gene Expression Omnibus (GEO, http://www.ncbi.nlm.nih.gov/geo/) database under the series accession GSE50033. *MIR*8788 was submitted to miRBase (http://www.mirbase.org/).

## Supporting Information

Figure S1 ***MIR*** **8788 processing in** ***P. infestans*** **,** ***P. ramorum*** **and** ***P. sojae*** **.** *MIR*8788 foldback diagrams for *P. infestans* (A), *P. ramorum* (B) and *P. sojae* (C). miR8788 guide strands and miR8788* strands are highlighted in each foldback. Proportion of small RNA reads for the entire foldback are plotted as stacked bar graphs. Small RNAs are color coded by size.
(EPS)

File S1 **Supplemental tables for small RNA-generating segments, genes and inverted repeats.**
(XLSX)

File S2 **Manually annotated gene sequences.**
(DOCX)
