# Supplementary material for: Phytophthora Have Distinct Endogenous Small RNA Populations That Include Short Interfering and microRNAs
Source: PLoS One. 2013 Oct 21;8(10):e77181. doi: 10.1371/journal.pone.0077181 (PMC3804510; doi:10.1371/journal.pone.0077181)
Supplement: File S2 — Manually annotated gene sequences. (DOCX) [file pone.0077181.s003.docx]

**MANUALLY ANNOTATED GENE SEQUENCES**

**Genes manually annotated with GENSCAN and FGENESH**

Key:

CDS

>PrRdr1 | scaffold_67:79140-87321 -

ATGACTGTTACCGCCAGCGCCAACGTGGCGTGGGGCGTCGCCTGCAAGCGATGCGACGCACTGGTGGCTCGTCACTCGCAGCTCTCGTTCCTCATGGAGAACTCGACGGCGATCCACTTGACGCTCGACAAGACGCTGCTGCACGACGAGCAGCGCGAAGCGCAGCCCCAAGCGGAGACGGGCGAGAGCTGGCGAGTGTACAGCCACGCGTACGTGAAGCCCGAGATCGCGTTGGTCAAGATCCGCACAACCAACGCGTACAAGAGGGAGCGGCTGCCCTTCGAGTTGTGCTGCGTTAAATGCGATGCAAAAGTTGCGTCCGAGGGGTTCCTGGACGAGCTGGACGAGCCGATGCTGCTCTTGGACTCGAAGGCGTGTGCCTGTGTGCTGGACAATCAGACGCCCTACGGGATGGCGGGGGGTACGAAGGCCAGGAAGTGGGGAGTGATCCTGAGGCAGCTGCAGGAGTTGAAGTTGCCGTTTAGGATTCAGAAACTGCAGGAGCTGATTGCTGCAGGGGGTGTTGAAGAGGAAAAACGGGAGAAGGAGGGCGAGAAGGAGGAGGTGGTGGTGCCGGTGGTGCACCCGACGGAAGCTTCCATTCGCAGTCATTTTAGTCCGAGAGCGAAGATGTCGCAATTGAGACGGTACCAGGTGGAGTTGACGTTGTCAGCGTTGCTGGAGAATACTATTGTGTATCTACCAACCGgtacgaggtgtatataattgacgggaaagtggagatttgtgaacttattgcttggctatgcttgtagGGTGTGGGAAGACTTTGGTGGCGATTAAGGTGATGGAAGAGATGAGGCATCTCAATCCCGACAAATTGGTGGTGTTTATCGTACCGACAGGACCGTTGGTGTCGCAGCAAGCCGGTTATATCCGACGAGAATCAAATTTCGAGGTGTCTGGATTATCGGGGCAACACAGCCGTACGGCGGCGGCGACCAGCGATCCAATTGAGATCGGTGGAGAGACAGATGCTATTGTGGTGACGCCGCAGTACTTTTTGAATCTTATGTTCAACGGCCGCACCAAAATCACAGATTACAGCACGGTGGTGTTTGATGAAGCACACCACGCGACTGGAAGTCACCCGTACTGTGAACTTCTGAAAAATATTGCTACGGTAGACCTCCAGGCTCGCCCACACATTCTTGCACTGACGGCTTCACCGTTTGGTGAATCAGTACGTGAAACATCGGGGCAGATAGCTCTGAACAAGCTTGCCAAAGCATTCAACGCCTCAGTAAATTCCCCTACAATCGCCGCGGACGATTTGGAATCGAGTTTTATCCCGAAGGAGGCGGAATGGGTTACTGTGGAGGAAAGTGAACCAGAGCAAAAACTCCGGGAGGGTATCAAGCGCTACATTGAGTCATTTTATGCGAAAATGTCGAGGCTAATGGGCGGCCAAGTTATGCCTTTCGAGGAAAATTATGACACGAATGACATGGAGCTGTCCCAATTCCTTGCGAAGCTGCGACTTTTACGTGTAGAAGCTGAGAAGAAGGCAATGGGGAACAGCAATGGTGTCGAGATGACAGGCATTGAAAAGAGCAAGCGCCCGCTGTCTGACCTGGAACTGATTCTTGAGCACCTACAAAAGGTGGCGTCGAGCTTTTACAGCTTGAGTATCCATGGCGCAGGTGCCGTTGCTGAGTCACTGCGGCGGATTTTCGGTGATTTGGAAGCAGCATCTTCCAAAAAGGGGAAACGCACATTTGCGCTTATTAGGTCGGCATACTGCTACACAATGAGCCCAGCATTAATCAATTTCCCGCTAGAGACCTTTAGTGGGGCCGACATCTCGAATCGTGTGCGCCTAGTCGCGGAGTGCATTCAGGACACTAACTTCGATCATGATTCTCGTGCAATCGTATTTGTCCGGCGCCGCAAAACTGCAATTCTGCTAGCGAAAGCAATGGAAGCATTGGATGTGTTACGTGAGCTTAACCCCACACGTTTTGTTGGACATAACTCTTATGAAGGAATGTCGTGGGAAGAGGAACAAAGGCCGACATTGGACCGCTTTCGACAAGGCCGCATCCGACTATTGGTTGCGACTAATGTATTGGAAGAAGGGCTGGACGTGCCTGAATGTTCGCTTGTTATTCAATTTGACGGAGTCGTTGGAGTCACGAGCCTTATTCAGAGTCGCGGTCGAGCACGTCACCGGGACAGCAGATTCATCATCTTCTGTTCTGCTGTAGGAATGGAGCGCAACCAACGCCTCGTGGAAAATGAAAAGCGGTTGGTGGCGATGGCTAATACAGCTGCCTCAAAACTAGAGTCCAAGGTTGCCGTGGAAAGGTTGATGAATGAGTACCCAGAAGAACTTGTTCCTAATACTGCAGTGAACACAGTTGGAGAGATGACGTTGCCACTGCGCGCGGATTTTGATTCGATGGCTGATAAAGTATACAACATTGTCCTAGGTGGAGTGTTTAAGGGAGCGAACAAGCAGCTACAAATGTCGATATTGGAAAGGGTAAGCACATTTGCCTCGGTTAGAATAGCAGACCAGCGTATCGGTTTGTGTACTCTCGCGGCACCTCCAAGCCAAGATATTTATGTTGCGTATAACATGCTATCGTCTGGTATCGATTTTTTCGTGGAGGCTGAATCATTCTGGATGAGGTTTGAAAGTGATGATATGGAGAGTGCAGCGAGTGGAAACTTTGCGGAGAATATTGCCGACGTGGGACTTCGCCGAGGATTAATGACCCCTGCTGGTGCTTTTACGGAGCTTGAGAGTTTTGGTAACACTGGTGGCCTAGAGATGGCAGCAGACGGCTTGATTGCATCGTTTGGTTACCGCCGTATAGAATTTGACTTGCGAGCGCTGTGTGAGCCACGAGTGTGGTTTGATTTGACTTCGACTGATATCGCTGCCGCGGTTTACCTCTCTTTTCGGCTCCCTCCTTCTTTTTACATTGGCGATGACCGTGAATGCAAGAGATGCCAGTCACAAGCACTCGTCTACTGTTTAATATTACCTCACTCCGACAATTCGAAGTCTCATATTTGGAATCTTCGCGTGTTCTTCTCAAAGCTCGGTGTCGAAGTACGTGAAACTAAGATCCCAGTGACAAACAAACAAACGGTTGCCTTGACTCAGCGCCACTTTACTTTTCCAGTGGCTTATGCTCTGCAGTGTCTACTTTCCGATTGTAGTCATTTCACGAATGGGTTCCTCCCGCCCGAATTTTTTGATGCGCTCACAGAACTTGATGAAAAAGTTCAAGAAAAGGCTCTCCTATCATTCCACGCACACCCTGGTCGCACCAACATTGCGGATCAATTTTCGGCTTATTTACATGACGAGCGCCCGATGCTAGAGGTACTTTGCTCCAGTTCGCACCTCGACGTGAGTGCCAGTGTCTACAAAGTCATTGTGACACCGTCTCGGATTGTCTACTGCCCGCCTGACCCAGCTCCAAGCAATCGGGTGTTTCGCCACTGGGGATCAGAAAACTTTATGTACGTGTACTTCCGCGATGACAATATGGAACGTTTGGACTACACGTCCGGGACGATTCTGGAGCGAGTGCGGAATGTTTTGAATGCGGGGATTGAAGTCCCACATGTTAAAGGAGGCCATAAGTTTCGCTTTCTTGGTTGCTCGCTTAGTCAGGTGCGCAACAGCAGCGCAATATTCACGTTCCTTGACCACAATAAAGTTCGTTCGTGGATTGGAGATCTTTCGCAATTGGCGTCTCCAGCAAAATATCTGAAGCGTCTCGGCCAGGCATTTTCCTCTACCAAAGAGACTTTCCGAGTCAACCAGAGTGTACTCGATTATCCGGTCAAAGACATTGAGAACGAGCTGTACACGTTCACAGATGGGTGCGGTGAGATTGCCCTCATTGGAGCAGAGAGCATCAAAGAGACTCTACATCTCGGATACACCCCATCTGCTTTCCAGATCCGTCTAGGAGGTGCAAAAGGTGTGTTGGTGGTATCTGATCTTGCCAATACGAATGTGAGCCGTGAAGATAGCGTGGTACTGCGTGAAAGTATGTCAAAATTCAAGTCCAATCACACTATGCTTGAAATAGTGGCGTGCGCAGGAAAATCTGAGGCTTACCTCACGCGTCAAAGCGTGCTGATATTGAACGATCTCGGGATTAACGAAGAAGTATTTCTGGAGATGCAAGAAGAGTTTTTGAGTGACCTACGCTCATTCATCGCGTCGAATGATGGTGCTTATTTCGAGCTAAAGGGTGTGTTACCGCCAAGCATTATTTGGTCAGTCGACGTGTTGATCGGTCAGCTTGGTGTGAAGGTTCTGGTTGATGACTTTCTATCGTCGCTGGCTAGAACCATTTATCATTACCGTCTCGCGAACACGGTGATGCGTGCACGAATACCCATCGCAAAAGGGCGTACATTGATGGGTGTGGCAGATTTCACAGGGACGTTGGCGTACGGCGAAGTGTTTGTTCAGTACTCTGAGACTGACGAAGATACGGGGATCGATCGCGCTGTTACTCTGGATAGCATCGATGTTGCTGTTCATCGTAGTCCGTGTCACCACCCTGGCGATATTCGGGTTCTTCGCTGCAGAGCAGATGTGCCTCTGCAACTACGTCAGCTAAAAGACTGTATTGTGTTTCCTTCCCAGGGTCCGAGGCCCCATCCTGACGAATGTACAGGTGGAGATTTGGACGGCGACATGTTCGTGGTCATTTGGGATAAACGCTTGATTCCATCTAGAGCTCAAGTTCAGGAGCCTATGACTTTTGACGAGGAATCTGGAGGTGATACCAGTCCCCGGGGTGGGGAGTATCACCAGTCTACTGACGATGAAGGGCTAGTTGATTTTTACGTGCACTCTATCCAAGACGATATCTTAGGTGTTGCTTCAAATGCTCACCTAGCGTTGTGCGATGCGTCACGTGGTGGAAGCTTTGGCGAGAATGCCAAGATCCTTGCACAAATTTGCTCAAAGCAAGTGGATAGTCTCCGGTCCGAGGCGGACTTGGAAATTGTGCGTAATCTGGCGCCCAAGAGTTATCCAGACTTTATGCAGAACAAGGATAAACCGTCATACCCGTCCAGCAAGGTGTTGGGCAAGATGTTTCGTCGCTGCAAGGCCATCTTTGATACAACGATGACAAAAAATGTGACTCAAATGCCGATGCGTGACGACCAGTTCCTGACGACAGGCTACACTGACTACCTAAATCACGCCAGAACGTTATATCGTCAGTACAAACTACGTCTCAGGGCCTTATTGCTGATGTCAGGAGCACAAACAGAGGCGGAGCTCGCTACTGGTATGATTGTGGATCCACAAAGTGTCTACAAGGCTGATTATTTCCGTTTCGGTGAGCAATGTAAGGACGCATTTTATGCTTTCCAGACGTCGTTTCGAAGTCAATTCGATAGTGACACCTCCGCAATGGCACCTGGTGAAAACCTCAAGGTTGCGGCGGCGTGCTATTTTGTCGCTTACGACGATTCTGACGCAGCGACACGCAGTTTAAGCTTTCCATGGATCGTAATCGATTTGCTCACGACCATCAAGAAAGCTAACATTGGTAAAGATTACTACCAACTGTGGAACCCCGTTCGATTATCGGCATACGCTGAAACAATTCCAAAGCTGCAATTGTGCATATTGGCCGAGATCACAGCAAACACCGATGAGTTGCTGGCAGACTTGTTTGATCGATTGGTTGCAGTGAGTTCATTGCGCTCAGTGATGCCTAGAACTCTCAAGAAGAAAGAGCTGGACCTCATTCTTTTCGGCTCTTCTGGACTACTAACGTTTGAAAAGCAATCTGACTTGGATGTGATGGTTCGTTGCACAACTTCACGCGGCTCACTAAAGGCAATTGCGAAGGCCCTGGCGGGATCCTATGGCAACATTGATTTGAAAGATGATATCCGCGTGCCACTTTTGTCTTTTTCATTTGACCAGTGGTCTGTCGAAATGTGTAAGCTTTCTAACGGACCAGTGAAAACGCGCTTGTTTAGGACATACATGGAGAGATACAACTTTTTCTGGCCGTGTGTATACTTCTTGGTGCGTTGGGGCAAGTGCGTCGGAGTGATTCGACGACGTTCAGGCGGTGGTCATGACATGTTTTCGCCCACGGGATTCATTTGGCTATTTTTGCGGTTTTGCACGGAGCATAATTTCGTGAACCCGATCAGCGCTGGTACTATCACGTTGAAGGACATTCTGAAGACCAACGATATTGATACGGAGATTTCATTTTGGACTGGGCTGCTTACTCGCTTGGTGTCCGGAAAAGAGGACGTATCCGCGCTTTCAGCGGCTGATGTCCTCCTTTCATTCTTCTCATATTATGCAAAATTATCCACACCTGATCGTGATTATGGATTTATAGACCCGCTCGATCCGGAGAACGACACCCAATTGGAACCCGAAGCGGTCACGCTGTTTCGGTCTGAGTGCCACATCGCGCTTCACCAGTTAGTGATCGTGCAGGGCGACATCAAGTATTTGTTGACGCATCGAGAAGAACAGTCGAGCAGAATCACACTCAGTCACGCGCTGAGTACACGCATTCACGCGGCGAAGGATTTTCTCTCTCGCAAGATCTTGTTCGAAACGAAAGCAAACAGCTCGACGCGTTTGAGTTTCAAATTTCATCCAAACTCCCTGCGTTCCGACTTGTACGTAGCTGAAATCATCGGACCTGGTGACGCAGTCCAGCGCATCGAAGAGCATATACGTGTGATCGAACAGGAGCTCGGGGCACGCATGCCATACCGCTCGAACCGGAATTTCCATCACGAAGGTTCGAGCCTGCTGCTATTCGAGGGAGCTGAATCCCAACACGAAAACATTGGCTTCCAGGATTATTTTGGAGAGCGGCACGGCGATCACATAAGGGATCCATTGCATCAAGCGCACTTGATTTGCTTTATGAACGGACGTCAGTGGTATGAGCATGCAGCCGCAACATTTTGCGCCAAGTTTATCCAGCAAATGATCAAACTGTCACGTTACGAACATTTGTATCCGGGTGCATCGGAGGCGAAAGCGTTTGTGCGCTTCGGACACCACTATTTGATAAACTTGCCACGCTCGTTCGCCCAGGAGACGATAATGCTGGCAAGTATCAAGAAGTTGGAAGACGAGTTCGAGCGAGGCAGAACTGCGCGCGAGCTATATGAGGCCGTTTTGATCGCGAAGCAGAAGCAGCGCGCTCAAGCGGAGGTTGAGAACATGATGAATCAAAATGATATACCACCTGAAGTGAACAACCAGGAAGCGGAGGGTGTTTGGGGAGAGTATTCTGGCGAAGAGCAAGATGGATCGGAAGCTGATGCGGATGAGGGCGAGCTCGACTgtgatggtggcggtggtggtggcggtgCGAGACAAAGGCGACGCCAGCGGCGGCCACGGATGAGCTTGCAGAAAGCAGATAAGGTTGCGCGAGGAGACAAGGGGGTAACGCATTCCTTCTACTCGATGATTGAGCCACAGCACACGCCATGGACAAAGGAGTACGCGGAGAAACGTCTGGGCATGACATTAGTCGAGCAAGAAGAATCCGATACGTACCAGGTATCCATAGTGCATCAGTCCTTCGAGTACAACATCAGGCTGACGTCTGATTTGCAACTCGTGAAGATCAAAACACGCCCGTCACGGTGGTTTAGCGCGACTCTAAAGATGCGGCAAGAGATGGATGATGAAAGTCACATGATGGACTTAACGCCGGACGTTCGCTTTTACGTGTCGACCACGACTGATATGCCCAAAACGGACAGTCTGTACGTGAAGCTGTCGACCTGCTGCGAGAATGCACCCGATGGTCGCGGGATCATTGAATTCTGTGACGACGAGACGCGCGACAAAGTACGCATTAGTCGGTACTTGGTTGATGCTGGCGAATCATCTGCGTGTATCGGGACGGTGAGGCATGTGCGCGGTACTAAGTACTATAACTCGGAGACGGAAACGCAGCTATCATTGATGCATATCCGTGAGTTTACTATTCCTGACCGGAACCCCCAGGATGGTTTTATGAGTGTGCGTGATAAAGTGGAGGCCGAATTCCTCCTTCCGCCGTTGACACCGGAGCGCCGCCTTCAGCCAACTTTCGCGCGTGGTTTCCTGACCACAGGTATGGAGTTTGTTGACTTTTTGCGTACCCAGGCAGACATGGCGATGCCTTAG

>PrAgo3 | scaffold_18:380881-384807 -

ATGAGCTACAGCGGAGGTGGCGGCGGCGGCTACGGCGACCAGCGCGGGTACAGCTCGCGCGGTGGCGACCACGGCCGCGGCTACGAGGATCCGAATCGCCCGAGTAGCAGCGGCAGCGGCGGAGGATACGGAGATCCAAGAGGGCAACAAGGGTACGATCAGCGCGGCCAGCAGGGGTACGGCGATCAGCGCGGCCAGCAGGGGTACGGCGACCAGCGTGGCCAGCAGGGGTACGATCAGAGAGGAGGGTACGAGCAGCAAAGAGGGGGGTACGACTCCCGCGGTGGCGGCGAGTACAGCTCGCGATCGTCGGGGTACGACGACTACAGTCGCGGCGGCGGAGGGGGTTACGGTGCCTCTGGATCGGGGTCTCGGGGGTACGACAGCAGAGGGGGCGACTACGGCTCTGGCAGTGGGAGTTACGGTAGTGGTGGTTACGATGACCGCAGTCGAGGAGCATCGTACGGCAGCGGGGGCTACGGAGGGTCGGGgtacgacgatcgtggagggcacggcagctcttctgggggatacgcaggatcatcaagttacgatagtcgaggtggctatgcagcctctgcgtcctctggaggttatgatcagAGCCGCAGTGGATATGATGACCGTGGTTATGGCGGAGGCAGTGGAAGCTATGGCAGTGGGGGAGGCGGCGGCGGACATGACGACCGCTCCCGAGGGTATGGAGGAGGTGGCCATGATGACCGCTCGCGCGGATATGGCAGCCGGGATGACCGCGGATATGGTGGTGGAGCGGCTGCTGGTGGCGGCCGTGATGATCGTGGATACGGTGCTAGCAGTGGCGGCGATCGTGGCTATGGTGGTGGTGGAGATCGTGGAGGTGATCGTGGCTACGGACGCCGCGATGATCGTGGAGGTGGTGATCGCCGTGGAGGAGATCGTGGATACGGTGGTAGCAGTGGCGGCGACCGCGGAGGTGACCGTGGAGGTGACCGTGGAGGTGACCGTGGAGGTGACCGTGGAGGAGATCGTGGATACGGCGGTGGAGGCCGCGGTGGCGGTCGCTCTGGAGGTGGTGGGGGTCGCGGTGGCCGCGGAGAGTCAGGCCCCGgtgttggcacggtgactggctccgggtatgagactttgggtgatcccgtggctgacccgcgtctccagGACGAGTGGACTGGTCGCGCGGGCGTGCGCGAAGTAAGCGCTGAAGACCCGGAGCTCCAGCAGGAGTTTCTTATTTGTCGCCGCCCGGGCGTCGCCAGAGGTGGCAAATCGATGCAGTTGAGTGTGAACTACTTCGGTGTCTCGCTCGACACGGTTCCAGCAGAAATTTTCAAATACCACGTGGATGTCGAGCGCTCGCCTGACCTCGCAGCAGACTCGAAGTACGGTCCTTCTGGCGGTACACCTGGAGACCAAAAGGATGAGACCATGGGTGACGAACCAAAGCAAGAGGGTAAAGACGAAAAAGAGGACAAGGACGTGGAAATGAGCGACGTGTCTGCACCTCCTAAACGTGAACAGCGCCCGGAACGACCCCTTCCACGCGCTCTGGTTCGCAACGTGATCAACGCTGCTCTCCGTCAATACGAGACTGAGTTTGGTGGCATCCGCGTTGTGCATGACGGTATGTCCGCCATGTATGCGCCTGCTATGATACCGTGGGAAGCGCAAACCAAGACTTTCGTCGACGTTAACCCGGACGGCCCCAGTCCGACCCCTCCGCCTCCTCCGCCACCAGCTGCAGGTGATGCGCCTCGCCGCCCGTTCCGAGGCCCTCGCACATTTGTCGTGAAGATCAAGCTCGCCGAGACGATCTCGACAAGTTCACTTACGGATTACTATTCCAACCCGGATGTGAATGTTATGCCGGTCCTCCAGGCGTTGGATGTGGCTGCACGTCATCTTGGAGCTCAGCGCTTGATCGCTGTGGGCCGCAACTTTTTCTCCATGAAAAAAACGTTCCCGCTCAAGGGTGGCAAAGAACTGTGTTGGGGTTACCACCAAGCTATTCGTATTGCCGACCGTAAACTGCTGATGAATGTCGATCAGGCGGCCACGGTGTTTTATGCTCCTAAGGAGCTGATGGAGCTTGTATTGGCCGCCTTAAGCGCACGATCGCCAAACGATATTCGCGGTTTGTCTGACCGGGATGCCAAGGCTTTGGCTCGCGCGCTACGCAAGATCGAAGTCGTTCCTACCCACCGCAAGGATCGCAAACGTGCAATTTTCGGTATTAGTGCGCAACCGGCTAACTTGACTATGGTGAACATTAAGGGGGAGGAAATGTCTGTGGCTGATTACTTCAACAAGCGCTACAACGTGCAGTTGAGACATCCGCAGCTCCCGCCAGTGAACGTTGGCAGTAAGCGAGCAGGCAAGGAGAACTGGTTGCCGATGGAGTTGTGCGAAGTGGCACCTGGACAGCGCTGCGCAAACATTAACGAGTTGGATACTGCCGAAATTATCAAACAGACCAGTCAGCCGCCGCGCGCTCGCAAAGAAACTATCATTGACCAGGTGCGCCAGGCCGGCTTCGAGAACGACCCGTACCTGGCTGCATTTGGCATGAAGGTGGAGCAACGGCTTGAAGCAACCGATGCTCGTGTGATGGATCCGCCCGAAGTCCAGTATGCGAATGTATCGGAGCGGCCATCAGGAGGTCAATGGAACTTGAGGGACAAGCGTTTTGTGGAAGGCGCTACTCTCCGTAACTGGGGTGTCGTGATCACTGCCAACGTTGGCGAGCGTGATGTGCAGGGATTCGTTCGCAACTTGGTGGATATGGCTGGCAAGAGTGGATTGACGATTGAGGACAGCAACCCGCACATGATCCACATGGACCAGTACCGTGGTTCGCAGGTCGAAGAGCTCATGAAGATGTGCTTCAAGGAGCTGGAGTCGCGTAACAGGGGACCTCCGCAACTTATCATGGTGATCAAGCAAGACAAGGGCGTTGGGTCCTATGGCGATATCAAACGCATGTCCGACACGGTGTTGGGTATCCCCAGCCAATGCATTGTGTCCCAGAACGTTCGCAGCGCAAAGCCTCAGTACTGTGCTAATGTCTGCCTCAAGATTAACATGAAGCTGAGCGGAAAGAACTCGATTCTGCGTGAGGAACTACCGCTGGTGAGTACGGCTCCTACAATCATTATCGGCGCTGATGTGGAGCACCCGCGCTCGGGCATGGGCGGGCGGCCTTCGATCGCTTCTGTTGTGGCTTCGCTTGACCGCTACTCGGCAAAATATGTTGCTCGAGTGGCTGCGCAAAAGGCTTCCAGTGATATCCAGCTACTGCCGCACATGCTGCGCGACCTGTTCTTGGCGTATTACCAGAGCACGAACCGCAAGCCGGAACACGTCATCTACTACCGTGACGGTGTGAGCGAGGGGCAGTTCTATGATATTTTACAGACCGAAATGCGTGCGCTTCGCAAGGCCTTCAAGATGATTTCGGAAGGCTACAACCCACCTGTCACCTTCATCATTGTGAACAAGCGCCACCATATGCGTGCATTCCCGGTCAACCAGCGTGACGCTGATCGCAAGGGCAATGTGGTTCCTGGAACGGTGATCGACACTGGCATTGTCGACTCTCACCGCTTCGACTTCTTCCTGTACGGCCACAGCGGCATCCAGGGAACTAGTGTGCCCTGCCACTACACCGTGCTCCATGACGAAAACAAAATGTCGGCGGAGGACGTGCAGCGTCTGACGTACCATCTCGGGTACACGTTTGCACGCTGCACTCGCTCGGTATCCTTTGCCACGCCCGCGTACTACGCGCATCTGGCTGCTGGTCGCGCGCGCTTCTTCCTGAACGAAGGCTCCGACGGGGCGTCAACTGTAGGCTCGTTCAACTCGAACTCGTCCAACTTCGACTTCACGGAGCTGCACAACGACCTCAAGAACTGCATGTTTTTCATCTAG

>PrAgo7 | scaffold_104:81401-84799 +

ATGTCGTGCGACCAGTTCGATTGCTACAACACGAGCTATAGCAGCGGACGATGGACTTCCGAATATAACGAAGGGGGAGGCTACGGAAGGCCGAGCAGCATTTCGGAGAGCAGAGACTCAAGATCTTACGAACCAAGTGCTGGCAATGATGGTCGTGGAAACAGTGGCAGTCAACGTGATTCAGATAACGGTCGCTACGGATCGGGCAGCTACGGTAGTAGGAGTTATGACTCTCGACGGCACGATGATCGACGTTACAGCGCTCGTAGCATTCCTTACGGAGGTCATGGCACATGGCCATTTCGTGCCGACAACCCTGACGACGATAGATCTCCATACTACGCTCGAGGCCGTGATGCTGGCAACTACGGCGACTATGACGATAACTCACGTGGTTATGAAAGACATGACCGACAACGTCCGAGTTCCTTCCGTACAAATGGAGGTCGTCACGATGAAGGAGAAAGAAGCGGGCGTTTTGGCGATAGAGCGCGCAATCACGATACGTTCCGCCGTGGTGATGGAGGAGGCCGCCGTGGTCCTCGAGATGAAGGTGGCGCTGGTGCAGGTTTCGTTGCTGGGTCTGGATACGAGGAGGTTGGTAATCCCCTTTCCAACTTACTCGAAGTAGACGAATGGAGTGGCCGCGCCGGTGCTCGACATGTACGCGCTGACGACGCCGAACTTCAGCAAGATGTGGCGGTTTGTCATCGGCCCCATGTTGCTGAGAGAGGGAGGACTGTGCTGCTCAACATCAACTACTTTAACGTCTCAATCGACTCGGCGCTTCGAGAGATATTCAAGTATCATGTTACTGTGGAGCGTACAGCGTCGAGCTTGAGATATGGTGCTTCAAGTGGCGACCACCAGGATGGGGCTCCAAGTGCTGTTGGTGAAGATACTGGAGCGTCAGAGACTACGAGACAAGACCACCAAAATGATGTTGCAAGTGGTGCTCGCGATAGTACTGGCGCGTCAGAAATTACGACACAAGAACCACGACCAACACGACCGCTGCAGCGTTCATTGGTCCGAGACGTTATCAATGCCGCCCTTCGAAAGTTCGGCGAGGAGTTTGCTGGTGTACGTGTGGTTCATGACGGCATGTCGGCTCTCTACTCGCCTGTCATTCTGCCGTGGAGCTCAAAGGAGTTTGCTGATGTCGACTTGGGCGGCGTTGGCGTCAACTCGTTCGTGACGCCCAGTGAAGGTGCCGTCAGTGCATCTCGTCGTAGAGGCCCACGAACATTTGTTGTAAAGGTGAACCTTGTGGAAACAATCTCTCTTTCGACTTTGGAGGACTTCTACTCCGATGCAATGGTGAATGTCATGCCCGTGCTGCAAGCTCTGGATGTTGTTGTCCGCCATCTCGCAGCGCAACGCTTGGTCGCGGTAGGGTCCGAGTTTTACAGCTTGAAGAAAACCCACACACTCAATGCCGGCAAAGAGCTGTGCTGGGGGTACCATCAAGCGATCCACGCCGCAGAGCGCAAACTGCTCGTGAATGTTGACCAAGCGACGGGGGTCTTCTACTCCCCAGGCCCTCTCATGCGCCTGGTTACAGCTGCGTTGCGCGTACGTAGCCCTCGCGAAGTCCAGAGATTGTCGGACCGCCAGCTAAAGGAGCTAGCGCGTGCATTGCGAGACGTGGAGGTGATACCAACACACCGCAAGGACTGCAAACGTGCCATCTGCGGCGTAAGCGCCTTGTCTCCCCACCAGATATTTATGAACGCTAAGGGTAAGGAGATATCCATCGCTGGCTACTTCAGCGAGCGGTACGGCAAGCAGCTCGAATACCCGAGTCTGCCACTTGTTAACGTTGGCAGCAAACGCCCGGGAAAGGAGACCTGGTTACCAATTGAGCTTTGTACCGTAGCACCGGGCCAACATTGCGCCAGCTCTGAAGATGTGGATGCACCAGAGATCACCCGTTTGACTAGCCAGCCTCCCCAGACACGCCAAGCAAATATCCTGGAGCACGTACGACAAGCACGATTCGAACATGACCCATTCTTGGAGGCCTTTGGCATGAAAGTGGAGCAACGTTTGCAGCGAATTGAGGCTCGCGTAATGGACGGTCCGCACGTTCAGTACCAGAACgtatcagtgcatcgctcagacgggcaatggagcctcaatgacaaaacgtttgtcaaaggtgtccgtgtccgcaactagGGTGTGATCGTTCTTGCCGACGTATGGAATAATGAGGTGCGAAAGTTTCTGCAGACGTTGTGTGAGGTCGGGAATGGGCACGGATTGCCCTTCGAGAACACACGCCCCGAGTTCGTTCACCAGAGCGAAAACCGTGGAGTGGGCGTCGACGAATTGATGACCAAATGTTTTCGACAGCTTGAGAAGTGTCAAGCCGAACGCCGTGCAGGGCCTCCGCAGCTATTGCTCGTAATCCTGCCAGACACAAGTTCCTTTCTTTATGGAGATGTTAAGAGGACGTCGGACACTGTTTTGGGCATTGCCAGCCAATGCATTGCATCAAAGAACCTGCGCAAGGCGAACGCCGCCTTTTGCGCGAACGTGTGCTTAAAGATTAACATGCGGCTGAATGGCAAGAATGCGGTTCTGCGTGGGCCCCTCCCGCTGATAAGCACTGCACCCACGATCCTAGTTGGCGCCGACGTTGAGTACCCGCGGCCAAGTTCGGACTATCAGCCAGCAATTGCTGCTGTCGTGGCGTCAATGGACGCGTATTCGGCCCAGTACGCAGCACGGGTGGCTGCTCAAAAGACGAGCAGCGAAATTCAAAGGCTGCCGCACATGTTGCGGGAACTATTTCTTGCTTACTTTGAGAACACGAAGCGCAGACCAGAGCATGTCATGTATTATCGAGGTGGAGTGGGCAAGGGTGAAATGGTGGACATTCTTCAGGCGGAGTTGCGTGCCCTGCGTATGGCGTTCAAAATGATTTCTGAAAACTACAGCCCGCTGGTTACGTTTATCGTGGCAAACAAACGCCACCACACACGTGCGTTTCCGGTGAGTCCCCGTGACGGCGACAGGAAGGGAAATGTGAAGCCAGGCACTGTGGTCGACGCTGGAGTTGTTGACCCTCATCGGTTCGAGTTTTTTCTATGGGGGCACACGAGTTTACAGGGTACCAGCAAGCCGTGCCGGTACACGGTCTTGCACGATGAGAATAATATGTCGGCGGGGGACGTGCAGCAACTGACGTATCATCTCGGATACACGTTTTCGCGATCGACCCACTCCGTGTCGGTCGTCACTCCCGTCTACTATGCGAATGAAGCTGCTGCGCATGCTCGTCACTTTTTACGGGAGGAGCCAAgtggggaatctaccgacggcacaagccaaacaaccttcagctttgaaaaagtccacccaaacgttcttaaccgcatgtgcttcatttgagGATGGAAAGATGAAGCAAGACTGTTGCAGGCAATGGATTCTTCTGTTGGCTTGAGCTTTTTGGTGGGCGGGGTTGCTCTTCATTTCGATGGCAGCGAAATACTTCGAAGTAGTTTTCATGAGGACGAAGAAGATAAGgtaagattgaagtttttgattccgacttggattgtagccattttatttcccgataatgaacgcctgattacataatccaacctctctaacgtcataaacgataccaaacttgacggaagagaagcctccccgctggctcccgttcaaagggtggaaagcaaccaccacagtaaaactccccagctccgtagCGTTTCTCCGTTGCCATGGCCTCCATCGCCGCGTCGCCAGgtacagtctccccccccctctctccttccccctccaccgtcggccacctcactctcttgtgtctctctttatggcccgctccccccttgcagTCTACCCGAACGGGCTCAAGAAGCGCTCGCGATTGACCGAGTCGTACGATTTGTCGAGGAAGAAGCGCCAGCGCCAAGCCAGCAATGGCCAGGACGAGGCCGTCGATGAGACCGACGTGGCCAAGTACAGCCAGCGCCACGTCGAGTACTTTGAGCAGGTGAAGCAGGCCGAGATCGCCCGAATAAGGGCCGAGTACGAGCAGTTCATCATGAAGAAGGACGCCGAGTTCCAGCGGCTCGGCCAGCAGCTGCAGCACACACAGGAGCGTGTGGCCGCCCAGGCCAGCGATGTGGCCCGTCTCCATGGCGAGAACAAGCTGCTGAAACGTGCAGTGGCCATCCAAAACCAGCAAAAGGAGGAGGTTCAGCACGAGAACAGCGCGCTCAAACAGCTGGCCACGCAGGCCGCCGAGCACATGAAGCGCCTCGAGCAGGCCAACTACACGCTCAGGGTCCACCTGCAGACCAGCACCAGCGCGGGCAACGGCCACCACTACCAGCCGCCTGATGTGTACTAG

>PiDcl2 | wgs contig

acagaaacctgccagtcaaaggaagatgaatattacgatttttgctctgcagatgaccaccgacactttcttcacttgatctgtacagatccttttcatttgcccaaaacgtcttttctcatgcttgtcattggccaaaagctgagtttaatttattcattttacgtgctgcatttggttgggaataggcttggatacgtgcgaagcaATGGACGACGTTTCCATTGGCCTCGAGATCGAGAACGCGTCGTTCCTGGGCGCATCCATGGCCGGTACAACAGAGGAAGCCCCACCCGAACCGGTGCCGGTGCCGGTGCCAGCTCCAGCGTCAACGTTAGCTTCTCTCACTCCGTCGCCCACCCCTTTTGCAACAACGCCAAATCTTCCAGTTGCACCAGTTGCAAGCCCAACCGAAGAAAATCTGCGCGTGTCTTACCACTGCAAAGCCCTCAAATGCTTCCGTCTCCAATGGGAGGCTGAAAgtgagcatcaatactgactgcattgcacgtgacatacaatctcactggtctttgcattgctcgcttctgcagTGAACCCGCGATGCCGCCGCCAAACAACGATTGTGATCGAGAATGCTGTCTATATCTTTGACGGGTTTTATCTATTGTGTCATGGCGAACCACCTGAGGTGCCGAGGgtgagtcatctgatctaaagctgctgttgctcttgagttacgactaagtgcgtgctcaactgtctttgtagTTTGAGTACACGCGTGGTGGTGTCATGTGCAAGATCCACTTTACCCCTGAAGACCTTCCGTCGTCCGTGCGTGGGACGACTAGCAACGAGGTGGACGCTGATGTCAATGGGGAAGCTCATCGATCTTTCTGCGACTCACTGTTTCTGCTTCAGCGCTACGTGTTCCAACAATTGCTGGGTATACAACCTGCTCATTTATTTGGGAAGGTTTTCCCGGATCCAGCTCATGCTACGGGTGACGCATCGGAAGTCTGTGGTGACTATCATCTAGTCCCTCGCTTTATCGCCGTGAACGAGGAGCCATTGCTCCACGTTGAAGACTTACATTCGCCCAACCGCGTGGGCTATGCTATCGGACGATTGTTATCCGTGCAAAGGATTCTGGAATACCTCGAAAACAGTGCGAGATTTACTGTACGCGATTTTCTGGTACAGTTCGATCACCGCGAGTTAGTGGACTGCGTTGTTTCGACTCGTATCAACGGCCAGCCAAGTGCGTTCCGTATCGACGAACTTGTTATGGAGACGCAAGAGGCCGAAGACGGAAAGCCTTCATCTGCCGAGAAGAACGGCATCGTTACAAAGGATGGACATCATGTTGTTCGAGCGATAGCTCACCGCAGTGAAGGAGCACGCAGCTTTGACGGAGCCAAGACGAAGAAGAAGATCCGCAAATGGAAAGAGCAAGGAGAGTTTTTACAGCTATCACCGGAGGTTGAACCTCTCTCCCATGAAGTACGTCCCGGAGAATGCCATCTTACAGGTATTCGCACTGATCTTTTCGAGGTTGGAAGCGCCGTGCCGATGGTGCTTAAGTATGTGCGGCATTTCAATTTACTGAACAGTTTTGGGACCACAATGGGTCTCACATTCACGGACAAGACGCTGCTACGTCAAGCGTTCACTCACGGCTCGTACATCGACGTGGGGATGCAGAACGTGAACACTGTCGAAGCCACACGGTCGCGTGTTCGCCTTGGACACGTGTTTGAGAACACTGTGTCGCTAAAGAGATCGCGTAGTATGTTGCTGGAAGGAGACACACGTATTCCTAGTAAGGCAGCAGGGAATAACGCGTCGCAACTACGACAAGTAGCAGAGAAACACTTGTCTGGAGATTTCAAGGAGGAATTCCACTCCCGGTATCTGTGTCCTTACGAACGTCTGGAGTTTTTGGGTGATGCCGTGCTTGGCTTTCTTGTGGCATCGTCGGCGTTTCTTAAGTTGCCCGAAGCTGATGAAGGATTCCTTCACCAGACTCGAGTTGACATTGTGAACAATGAAAATCTCGGGAAGATGGCGAAAACTGCGAACTTTGAGTCACTTTTGCTGACTGCATTCGACTTGGCCAAGTTGAATGAAGATATTAAGGCGAAAATCACGGCAGATTGCTTCGAAGCACTTCTAGGCGCACTCTATAAAGACCAAGGCATTGCTCCATGTCGAGAATTACTCGGTAAACTTATGGATATGCACGACCCAGAACTGCGAGAGCTGTGCTATTTATCAACCGATGAAGTCGTTGCCCACGCGAAAAAATATGTGGAGAAAGACCGTGCTGATATCAAGAAATGGAGCAAATATACCAGCACTCGCTTGCTGCACCGTCGCTTTGCTGCACGCTCGGGCGTCGATATCTCCAACACACACTTGTGGCTACAAGCATTCACTCACGCATCGTTCCAGAAGCCTCAGATTGGAGACGATGAGTTCATCGGACATGAACCAAGTTACGAACGTATCGAGTTTCTGGGCGACGCAGTGTTGCAGCTACTGTCCTCCGAGTTCCTTGTTGATGCATTCCCGTATCATCAGGAGCATCTTTTGACCCAAGTACGCTCTTCGCTAGTGAAGAACAAGAAGTTGGCGATCGTTGCTCGTAAAGCCGGATACGAGGAGTTCATCCGCTTAGGCAAGCTTGTCAAGGAGAACGGCAATTTGTACGTTGAAGACGTACTAGCTGATGTGTTTGAAGCTACACTTGGTGCTGTGTACATGGAGAACCCGGCAGATTTAGAAAAAGTTCGCTCGATCCTCGAACACTTGCTGTTCCCTCGACTCACGGAGGCTATTCGACGTCGACAGTGGATGAATCCTCGCAAGGTGTTCATGCACTACATCGCGCAATGGAGTCGTACCAGCATGCGGCAGATTCAGTGTCAGTTTAAGAACATTCAAGTTCCAAGCTCAACTACACGACCAAATATCTTGGTGCCCAAGGATAATGAAGGAGCTAAGAGAAAAAAGGCTAAGACTAATGCGCCTCCAGGACACGCGGTGGCTCTTTATGTGAACGGCTTTCAAGTGTCTCGCGCGGTCGGACGCACCATTGCCGCAGCTCAAGATGCAGCCTGCAAGAAAGCTCTCATGCTTTACGGTGTCCGACTGCATGACGACTAAgatgaaggaataatgctaccagcagcaaagattaagtaggcaacattaagctggtagtggtaagagttctttgtatttgtaagcgagtacttcgtgtatgccgaacgtgaactaaccgagctgagtgctgtctatccgagacttcgttctctttagtcctttgcctttttcgatgaggtcggtgggggaccgggaggaagcggtggcttctttccaaaaggaaggggaggaggcttgggcttggatgccccctccgctgtcggttgaggtccacgaggtttgtcctcgtccttgccatcccgtcgctgattcttcttctgtccgccgagcatcttcgcggctacagggtcattcttgccgtagaatcggtctttgatgttctgcttcgccagcgggtcatccttatcgcgtggcatctcgtgcagatagggacactcgtcgcctcgattacattcccctcgagcaaagaatgaacacaagtgtgcacggttgcgtttgtagtacggctcacgacgtgccatacgcagcagagcgttattagccgagcccgacgctttgccatatgctgacacggctccctcctgctccaacatacgactatgctgttgtgagaaccactcgcgattggcgtcgctctcaggtacgttgacagctaactggtcgccgccttcttccctggccaagaccgtgtcgcgcacctgcaccggcagatgatactgcagatctagcacgcaggtctgacatacattcttcatgccgcgcacaagtctggcacactcggtctttcttgtaacgcgcccccttgccggtttccagcgaaaaacagtgaa

***P. infestans* whole-genome shotgun reads from the NCBI Trace Archive that match the *PiDcl*2 locus**

[1320689468](http://www.ncbi.nlm.nih.gov/Traces/trace.cgi?&cmd=retrieve&val=1320689468&retrieve=Submit); [1302821941](http://www.ncbi.nlm.nih.gov/Traces/trace.cgi?&cmd=retrieve&val=1302821941&retrieve=Submit); [1314306713](http://www.ncbi.nlm.nih.gov/Traces/trace.cgi?&cmd=retrieve&val=1314306713&retrieve=Submit); [1312068582](http://www.ncbi.nlm.nih.gov/Traces/trace.cgi?&cmd=retrieve&val=1312068582&retrieve=Submit); [1311174022](http://www.ncbi.nlm.nih.gov/Traces/trace.cgi?&cmd=retrieve&val=1311174022&retrieve=Submit); [1313582598](http://www.ncbi.nlm.nih.gov/Traces/trace.cgi?&cmd=retrieve&val=1313582598&retrieve=Submit); [1314070700](http://www.ncbi.nlm.nih.gov/Traces/trace.cgi?&cmd=retrieve&val=1314070700&retrieve=Submit); [1320700397](http://www.ncbi.nlm.nih.gov/Traces/trace.cgi?&cmd=retrieve&val=1320700397&retrieve=Submit); [1317486100](http://www.ncbi.nlm.nih.gov/Traces/trace.cgi?&cmd=retrieve&val=1317486100&retrieve=Submit); [1151610365](http://www.ncbi.nlm.nih.gov/Traces/trace.cgi?&cmd=retrieve&val=1151610365&retrieve=Submit); [1183505687](http://www.ncbi.nlm.nih.gov/Traces/trace.cgi?&cmd=retrieve&val=1183505687&retrieve=Submit); [1311203574](http://www.ncbi.nlm.nih.gov/Traces/trace.cgi?&cmd=retrieve&val=1311203574&retrieve=Submit); [1322811780](http://www.ncbi.nlm.nih.gov/Traces/trace.cgi?&cmd=retrieve&val=1322811780&retrieve=Submit); [1320128384](http://www.ncbi.nlm.nih.gov/Traces/trace.cgi?&cmd=retrieve&val=1320128384&retrieve=Submit); [1311225208](http://www.ncbi.nlm.nih.gov/Traces/trace.cgi?&cmd=retrieve&val=1311225208&retrieve=Submit); [1317291790](http://www.ncbi.nlm.nih.gov/Traces/trace.cgi?&cmd=retrieve&val=1317291790&retrieve=Submit); [1319691624](http://www.ncbi.nlm.nih.gov/Traces/trace.cgi?&cmd=retrieve&val=1319691624&retrieve=Submit); [1320057338](http://www.ncbi.nlm.nih.gov/Traces/trace.cgi?&cmd=retrieve&val=1320057338&retrieve=Submit); [1369245275](http://www.ncbi.nlm.nih.gov/Traces/trace.cgi?&cmd=retrieve&val=1369245275&retrieve=Submit); [1312806356](http://www.ncbi.nlm.nih.gov/Traces/trace.cgi?&cmd=retrieve&val=1312806356&retrieve=Submit); [1313375287](http://www.ncbi.nlm.nih.gov/Traces/trace.cgi?&cmd=retrieve&val=1313375287&retrieve=Submit); [1314225760](http://www.ncbi.nlm.nih.gov/Traces/trace.cgi?&cmd=retrieve&val=1314225760&retrieve=Submit); [1310315148](http://www.ncbi.nlm.nih.gov/Traces/trace.cgi?&cmd=retrieve&val=1310315148&retrieve=Submit); [1312870273](http://www.ncbi.nlm.nih.gov/Traces/trace.cgi?&cmd=retrieve&val=1312870273&retrieve=Submit); [1313953242](http://www.ncbi.nlm.nih.gov/Traces/trace.cgi?&cmd=retrieve&val=1313953242&retrieve=Submit); [1304282359](http://www.ncbi.nlm.nih.gov/Traces/trace.cgi?&cmd=retrieve&val=1304282359&retrieve=Submit); [1376397240](http://www.ncbi.nlm.nih.gov/Traces/trace.cgi?&cmd=retrieve&val=1376397240&retrieve=Submit); [1311984303](http://www.ncbi.nlm.nih.gov/Traces/trace.cgi?&cmd=retrieve&val=1311984303&retrieve=Submit); [1324312713](http://www.ncbi.nlm.nih.gov/Traces/trace.cgi?&cmd=retrieve&val=1324312713&retrieve=Submit); [1384658500](http://www.ncbi.nlm.nih.gov/Traces/trace.cgi?&cmd=retrieve&val=1384658500&retrieve=Submit); [1316503440](http://www.ncbi.nlm.nih.gov/Traces/trace.cgi?&cmd=retrieve&val=1316503440&retrieve=Submit); [1324200912](http://www.ncbi.nlm.nih.gov/Traces/trace.cgi?&cmd=retrieve&val=1324200912&retrieve=Submit); [1317283442](http://www.ncbi.nlm.nih.gov/Traces/trace.cgi?&cmd=retrieve&val=1317283442&retrieve=Submit); [1313794137](http://www.ncbi.nlm.nih.gov/Traces/trace.cgi?&cmd=retrieve&val=1313794137&retrieve=Submit)

***MIRNA* foldback sequences**

Key:

**mature miRNA**

**mature miRNA***

>*pin-MIR*8788

GCGAGAGCTCGCCTCGC**CTACCAAGCGTACCAGCGCCG**CATTTCAGCACACGAGCGCTCTCTGGCTTAATGGTTAGCTTGCACTCGTTTGCTGAGTTGCA**GCGCTGGTTCGCTTGGTACGC**TAGGTGCACTCTGGC

>*psj-MIR*8788a

GCGAGAGTTCGCGTAGC**CTACCAAGCGTACCAATACCG**TAATTCAGCACACGAGTGCGTGAGGGTGGCGAGCCCCCCCCGCGCGCTCGTTTGCTGAGATGCA**GTGTTGGTTCGCTTGGTACGC**CCCGTGCACTCTTGC

>*psj-MIR*8788b

GCGAGAGTTCGCGTAGC**CTACCAAGCGTACCAATACCG**TAATTCAGCACACGAGTGCGTGAGGGTGGCGAGCCCCCCCCCCGCGCGCTCGTTTGCTGAGATGCA**GTGTTGGTTCGCTTGGTACGC**CCCGTGCACTCTTGC

>*pra-MIR*8788a

GCGAGAGTTCGCTTCGC**CTACCAAGCGTACCAACGCCG**CATTGTAGCAAACGAGGAGTCCAAGGTGGCGCAGCCTCTCGGGCCTCTCATTTGCTGCGCTGCA**GCGCTGGTTCGCTTGGTACGC**CGAGCGCACTCTTGC

>*pra-MIR*8788b

GCGAGAGTTCGCTTCGC**CTACCAAGCGTACCAACGCCG**CATTGTAGCAAACGAGGAGTCCAAGGTGGCGCAGCCTCTCGGGCCTCTCATTTGCTGCGCTGCA**GCGCTGGTTCGCTTGGTACGC**CGAGCGCACTCTTGC
